# The *epidermal bladder cell‐free* mutant of the salt‐tolerant quinoa challenges our understanding of halophyte crop salinity tolerance

**DOI:** 10.1111/nph.18420

**Published:** 2022-08-30

**Authors:** Max William Moog, Mai Duy Luu Trinh, Anton Frisgaard Nørrevang, Amalie Kofoed Bendtsen, Cuiwei Wang, Jeppe Thulin Østerberg, Sergey Shabala, Rainer Hedrich, Toni Wendt, Michael Palmgren

**Affiliations:** ^1^ Department of Plant and Environmental Sciences University of Copenhagen Thorvaldsensvej 40 DK‐1871 Frederiksberg C Denmark; ^2^ Carlsberg Research Laboratory J.C. Jacobsens Gade 4 DK‐1799 Copenhagen V Denmark; ^3^ Traitomic, Carlsberg A/S J.C. Jacobsens Gade 4 1799 Copenhagen V Denmark; ^4^ School of Land and Food University of Tasmania Hobart Tas. 7001 Australia; ^5^ International Research Centre for Environmental Membrane Biology Foshan University Foshan 528000 China; ^6^ School of Biological Science University of Western Australia Perth WA 6009 Australia; ^7^ Julius‐von‐Sachs‐Institut für Biowissenschaften, Biozentrum University of Würzburg D‐97082 Würzburg Germany

**Keywords:** abiotic stress, *Chenopodium quinoa*, epidermal bladder cells, quinoa, salt tolerance

## Abstract

- Halophytes tolerate high salinity levels that would kill conventional crops. Understanding salt tolerance mechanisms will provide clues for breeding salt‐tolerant plants. Many halophytes, such as quinoa (*Chenopodium quinoa*), are covered by a layer of epidermal bladder cells (EBCs) that are thought to mediate salt tolerance by serving as salt dumps.
- We isolated an *epidermal bladder cell‐free* (*ebcf*) quinoa mutant that completely lacked EBCs and was mutated in *REBC* and *REBC‐like1*. This mutant showed no loss of salt stress tolerance.
- When wild‐type quinoa plants were exposed to saline soil, EBCs accumulated potassium (K^+^) as the major cation, in quantities far exceeding those of sodium (Na^+^). Emerging leaves densely packed with EBCs had the lowest Na^+^ content, whereas old leaves with deflated EBCs served as Na^+^ sinks. When the leaves expanded, K^+^ was recycled from EBCs, resulting in turgor loss that led to a progressive deflation of EBCs.
- Our findings suggest that EBCs in young leaves serve as a K^+^‐powered hydrodynamic system that functions as a water sink for solute storage. Sodium ions accumulate within old leaves that subsequently wilt and are shed. This mechanism improves the survival of quinoa under high salinity conditions.

## Introduction

Soil salinity, affecting 25–30% of all irrigated land (Shahid *et al*., 2018), causes major crop losses world‐wide and is a growing problem, particularly in arid and semi‐arid countries that depend on irrigation for agriculture (Liu *et al*., 2020). In crops, salinity stress is associated with an increased uptake of sodium (Na^+^) and chloride (Cl^−^) and their accumulation to toxic levels in the plant. However, plants known as halophytes can complete their life cycle even in the presence of 200 mM NaCl and may even show improved growth in the presence of NaCl (Flowers & Colmer, 2008; Shabala, 2013). Among these plants are members of the order Caryophyllales, such as the Aizoaceae family, exemplified by the ice plant (*Mesembryanthemum crystallinum*), which can undergo crassulacean acid metabolism (CAM; Guan *et al*., 2020), and the Amaranthaceae family, which includes the ancient crop quinoa (*Chenopodium quinoa*; Orsini *et al*., 2011). Understanding the mechanisms by which these halophytes tolerate salt stress is key to breeding more salt‐tolerant crops and therefore is important for future food security.

A striking morphological feature of *M. crystallinum*, quinoa, and *c*. 50% of all halophytes, is the presence of so‐called epidermal bladder cells (EBCs), which cover the aerial surface of the plant and are particularly pronounced on the surfaces of young leaves (Shabala & Mackay, 2011). Epidermal bladder cells are modified trichomes that are globular in shape and > 100 times larger in volume than leaf cells (Böhm *et al*., 2018). The cell sap of single *M. crystallinum* EBCs can be removed with a glass capillary and contains up to 1 M each of Na^+^ and Cl^−^ (Adams *et al*., 1998). The ion concentrations in quinoa EBCs were not measured directly; instead, by comparing the ion content in the leaves of quinoa plants which possess EBCs to the ion content of leaves from which the EBCs were mechanically removed. It was calculated that the ion concentration values in quinoa EBCs (Na^+^, 850 mM; Cl^−^, 1 M; Kiani‐Pouya *et al*., 2017) might be similar to those observed in *M. crystallinum* EBCs. It has thus been suggested that the EBCs of both *M. crystallinum* and quinoa serve as ‘salt dumps’ (Kiani‐Pouya *et al*., 2017; Brownlee, 2018). Additionally, it has been suggested that EBCs store water and act as a ‘secondary epidermis’ that reduces transpiration (Agarie *et al*., 2007; Shabala & Mackay, 2011).

An argument in favour of the importance of EBCs for the salt tolerance of quinoa has come from the demonstration that removal (by brushing) of the EBCs from young quinoa plants appears to make them more sensitive to salinity, without altering other phenotypic characteristics (Kiani‐Pouya *et al*., 2017). Genome sequencing and analysis of the EBC transcriptome in quinoa (Jarvis *et al*., 2017; Böhm *et al*., 2018) revealed that many genes encoding cation transporters are highly expressed in these cells, as expected for salt‐accumulating cells, albeit expression of most of these genes was constitutive and not influenced by salinity stress (Böhm *et al*., 2018).

The genetics of EBC formation in quinoa has not been fully elucidated. Recently, Imamura *et al*. (2020) identified *REBC* (*AUR62039690*) as a quinoa gene involved in EBC formation, especially on the leaves. *Rebc* mutant plants still have EBCs but in much lower numbers, and they suffer from developmental defects at the apical meristem. *REBC* encodes a member of the family of WD‐40 domain‐containing proteins, which in Arabidopsis contribute to trichome development (Pattanaik *et al*., 2014) and in quinoa includes an additional member, *REBC‐like1*, the function of which is unknown.

We measured ion concentrations in the EBCs of quinoa and observed, to our surprise, that in response to salt stress they preferentially accumulate K^+^ and only small quantities of Na^+^. To study Na^+^ tolerance in more detail, we isolated an *epidermal* mutant that completely lacks EBCs (*epidermal bladder cell‐free*; *ebcf*). In the latter, both *REBC* and *REBC‐like1* were found to be mutated. *Ebcf* plants appeared completely healthy and, to our astonishment, were as salt tolerant as the wild‐type. Based on our new findings, we conclude that under the experimental conditions tested EBCs do not play a major role, if any, in the salt tolerance mechanism of quinoa.

## Materials and Methods

### Plant materials and growth conditions

The *Chenopodium quinoa* Willd. Cultivar 5206 (Titicaca) was used in this study. Unless otherwise stated, single seeds were sown in 2‐l pots containing standard potting soil (Pindstrup Substrate, Ryomgaard, Denmark) and were irrigated with regular tap water. Plants were either grown in a semi‐controlled glasshouse (in Copenhagen, Denmark), where the set conditions were influenced by the weather (conditions: a 16 h : 8 h, 20°C : 18°C, light : dark cycle; 35–98% relative humidity (RH); and 210 μE photosynthetically active radiation (PAR)), or in controlled growth chambers. Conditions in the growth chambers were as follows: a 16 h : 8 h, 22°C : 18°C, light : dark cycle; 60% RH; and 180 μE PAR.

Salt treatment started at 14 d after sowing (DAS), with NaCl concentrations gradually increasing from 12.5% (14 DAS) to 25% (16 DAS) and 50% (19 DAS), until a final concentration was reached at 21 DAS. Control plants were irrigated with regular tap water. To avoid positional effects within the glasshouse or growth chambers, the positions of the trays containing the plants were regularly changed.

### Generation of a mutant quinoa population for phenotypic screening

To generate a population of mutagenized quinoa, 5 kg of quinoa *M*_0_ seeds (cv Titicaca) (QuinoaQuality, https://www.quinoaquality.com/) was submerged in a 0.2% (v/v) solution of ethyl methanesulfonate (EMS) in water for 16 h at room temperature. Mutagenized seeds (*M*_1_) were then thoroughly rinsed in water and left to air‐dry for 24 h. *M*_1_ seeds were sown at a density of 6 kg ha^−1^ and grown to maturity under field conditions in Denmark during the 2019 field season. Seeds (*M*_2_) were harvested from 14 000 mature *M*_1_ plants. *M*_2_ seeds were threshed and cleaned, and a bulk seed population was sown for phenotypic screening. Seeds were sown at a density of 9 kg ha^−1^ over an area of 1000 m^2^ in a field in Denmark during the 2020 season. Phenotypic screening was performed by eye 2 months after sowing. A single plant (*M*_2_ generation) with no EBCs was identified, and its seeds (*M*_3_ generation) were harvested. As this plant could have been pollinated by wild‐type (WT) plants, *M*_3_ plants were grown and selfed under glasshouse conditions (at the University of Copenhagen, Denmark). In the offspring (*M*_4_), a plant (#4) that showed a complete loss of EBCs (*ebcf*) was selfed, and its seeds (*M*_5_) were then sown for further phenotypic analysis (see Supporting Information Fig. S1 for a schematic explanation).

### Genotypic analysis and backcrossing of *ebcf* mutants

The genomic sequences of *REBC* (*AUR62039690*) and *REBC‐like1* (*REBCL1*, *AUR62009292*) were identified in ChenopodiumDB (https://www.cbrc.kaust.edu.sa/chenopodiumdb/index.html) using a Blast search with XM_021859495 and XM_021883470 (Imamura *et al*., 2020) as query sequences, respectively. The *REBC* genomic sequence was amplified using the REBC_Fw (5′‐TGCATGCATATATACCCCCTAG‐3′) and REBC_Rv (5′‐ATTTAAATTCATATGTAATGGTGCAT‐3′) primers. The *REBC‐like1* genomic sequence was amplified using the REBClike1_Fw (5′‐CTAGCTCCCAATAACCTAGTTCCAA‐3′) and REBClike1_Rv (5′‐TACGAATTATACTTGTGATATCTGCCAA‐3′) primers. Polymerase chain reaction (PCR) products were purified and then sent to Eurofins (https://eurofinsgenomics.eu/) for sequence analysis or treated with *Bsp*EI restriction enzyme (New England Biolabs, Ipswich, MA, USA). Digested DNA products were analysed on a 1% agarose gel after electrophoresis.

For backcrossing, seeds of the *M*_5_ generation completely lacking EBCs were sown together with WT seeds. Right before flowering started, a mutant (*P*_0_) and a WT (*P*_0_) plant were covered together with a plastic bag. As soon as both plants were flowering, the flower buds were shaken regularly to distribute the pollen and increase the chance of crossing. Seeds produced by the EBC‐free mutant were sown and the *F*_1_ generation was screened for plants with EBCs, indicating a successful crossing event. These plants were then genotyped, and a plant was identified that carried a heterozygous mutation in both *REBC* and *REBC‐like1*. The offspring of this selfed plant (*F*_2_) were used for segregation analysis.

### Comparing the salinity response of wild‐type and *ebcf* plants

To test the growth response of *ebcf* plants (*M*_5_) to salinity, mutant and WT (cv. Titicaca) quinoa plants were grown in a controlled growth chamber. After 2 wk, the plants were watered with tap water with no NaCl (0 mM), 50 mM NaCl (moderate salt stress), or 400 mM NaCl (severe salt stress). Gas‐exchange, pulse‐amplitude‐modulation (PAM), and thermal image measurements were made on the young leaves after a full 4 wk of salt treatment (49 DAS). Samples were collected for ion and real‐time quantitative polymerase chain reaction (RT‐qPCR) analysis at the same time.

For ion analysis, measurements from young, intermediate, and old leaves from WT and *ebcf* plants were compared. The intermediate and old leaves of WT plants possessed only a few or no intact bladder cells. Leaves beginning from the first clearly defined leaf to the oldest leaf, excluding the cotyledons, were classified according to their relative position among all of the leaves on the plant, as follows: emerging (15%), young (30%), intermediate (55%), or old (80%) (see Fig. S2).

After 2 wk of controlled growth and an additional 5 wk of salt treatment (56 DAS), *ebcf* and WT plants were harvested, photographs were taken, and the fresh weights (FWs) and plant heights were determined. Dry weights (DWs) were measured after 4 d of drying at 70°C, beyond which no further weight reduction was observed.

In addition, *ebcf* and WT plants were grown from seed in a growth chamber under standard conditions for 2 wk. Plants were then either moved to a different growth chamber with additional UVB150 light bulbs (ExoTerra, Hagen, Germany) illuminating the plants from the top with 75 μW cm^−2^ or subjected to wind stress applied by a fan from above at 45° relative to the shoot (at a flow rate of 13 m^3^ min^−1^) for 4 wk. The shoot apices were then evaluated for damage.

### Salinity response and epidermal bladder cell brushing of different quinoa cultivars

In addition to the Titicaca cultivar (cv. 5206), five other quinoa cultivars, 144 (sensitive), 149 (tolerant), 189 (tolerant), 197 (sensitive), and 350 (sensitive), which were classified as tolerant or sensitive as indicated, according to Kiani‐Pouya *et al*. (2019), were grown in a glasshouse between 27 September and 15 November 2021 and analysed during this period (seeds obtained from IPK Gatersleben, Gatersleben, Germany). To investigate the role of EBCs in salt tolerance, EBCs were removed three times per week by gentle brushing with a cosmetic brush (brushed plants), as described by Kiani‐Pouya *et al*. (2017). To reduce the number of leaves that needed brushing, all side branches were removed on a regular basis. In addition to the brushed plants, plants were grown from which only the side branches were removed (nonbrushed plants). Treatment with 400 mM NaCl was conducted as described in the previous sub‐section and lasted for 5 wk in total.

At the end of the experiment, young leaves of nonbrushed plants with high EBC densities were collected and split into three parts for ion analysis. One longitudinal half of the leaf was kept as is, meaning that the EBCs were intact and present (nonscraped). The EBCs were carefully scraped off the other half with a spatula, leaving only the portion of the leaf without EBCs (scraped). The scraped half was then rinsed with MilliQ‐H_2_O to remove residual burst EBCs. The removed EBCs were kept as a third sample (EBC) (see Fig. S3a–d). The central midrib was excluded. By gently scraping the leaves, most of the EBCs could be removed from the leaf surface without damaging the leaf or the EBCs, resulting in a large number of intact EBCs for analysis (see Fig. S3c). For all samples, fresh and dry weights were recorded. Subsequently, photographs of plants were taken, and plants were harvested to determine their biomass.

### Detailed ion content analysis in epidermal bladder cells

For a detailed analysis of the ionic composition of EBCs, different experiments were conducted under glasshouse conditions. In one experiment, after quinoa plants were grown from seed under control conditions for 4 wk, the plants were irrigated with 300 mM NaCl solution. Epidermal bladder cells from young and emerging leaves were collected at different time points after salt application (0, 1, 4, 8, 24, 48, and 168 h (7 d)) (plant growth from 12 May to 16 June 2020). In another experiment, one set of quinoa seeds was irrigated with tap water and continued growing without any added salt. Another set was irrigated with 200 mM NaCl solution, and after 1 wk, the NaCl concentration was increased to 400 mM. Epidermal bladder cells were scraped from the plants at 12, 19, and 30 DAS. The 30 DAS EBCs (scraped from the underlying leaf portion) could be divided into EBCs of young and emerging leaves (plant growth from 17 May to 16 June 2020). In a third experiment, after 2 wk of growth under control conditions (0 mM NaCl), plants were irrigated with 400 mM NaCl for 6 wk. Leaf and EBC samples were taken from young and old leaves. While EBCs were scraped from young leaves, dry EBCs were collected by brushing from old leaves into a 1.5‐ml reaction tube with a cosmetic brush (Plant growth from 16 December 2021 to 10 February 2022).

### Ion analysis

Dry plant material was prepared and analysed as described previously (Munns *et al*., 2010). Briefly, nutrients were extracted from dried plant samples with 0.5 M HNO_3_. Concentrations were calculated using standards containing the analysed nutrient. The Na^+^ and K^+^ concentrations were measured using a PFP7 Flame Photometer (Jenway, Stone, UK). The Cl^−^ concentration was determined using a 926 Chloride Analyser (Sherwood Scientific, Cambridge, UK).

### Chl fluorescence characteristics

The state of the Chl fluorescence of excised and dark‐adapted leaves (15 min darkness, from growth chamber‐grown plants) was measured with an Imaging‐Pam‐Max/L (Walz, Effeltrich, Germany) and analysed with ImagingWinGigE v.2.47+ (Walz). The effective quantum yield of photosystem II (PSII) was determined, which was defined as *F*_v_/*F*_m_ = (*F*_m_ – *F*_0_)/*F*_m_, where *F*_v_ is the fluorescence emission from a dark‐adapted leaf and *F*_m_ is the maximal efficiency from a dark‐adapted leaf.

### Gas‐exchange measurements and thermal imaging

Gas‐exchange measurements were performed using a GFS‐3000 portable gas exchange fluorescence system (Walz). Young leaves were individually clamped into the measuring head, and measuring points were recorded after steady state values were achieved. For analysis of WT and *ebcf*, conditions were set to growth chamber conditions. The measuring gas was set to a flow rate of 750 μmol s^−1^ with 60% RH and 420 ppm CO_2_ at 23°C. A leaf area of 2.5 cm^2^ was illuminated with 180 μE from the 3041‐L LED light source (warm‐white LEDs; Walz). Values for assimilation and transpiration were provided by the GFS‐3000 and calculated according to Von Caemmerer & Farquhar (1981).

Thermal images were taken with an infrared camera (FLIR A35 sc; FLIR Systems AB, Täby, Sweden) and analysed using the ResearchIR software package (FLIR).

### Quantitative polymerase chain reaction analysis of *Chenopodium quinoa* HAK6, SOS1, HKT1.2, and NHX1.1

Total RNA was purified from leaves of WT and *ebcf* mutant plants using the Spectrum Plant Total RNA Kit (cat. no. STRN50‐1KT; Sigma) with an ‘on column’ DNase treatment step using an RNase‐Free DNase Set (cat. no. 79256; Qiagen). The subsequent cDNA synthesis was performed using a QuantiTect^®^ Reverse Transcription Kit (cat. no. 205313) from a starting concentration of 1 μg RNA.

Brilliant II SYBR^®^ Green QPCR Master Mix (cat. no. 600828; Agilent Technologies, Santa Clara, CA, USA) was used for qPCR, which was carried out using the Stratagene Mx3005P qPCR machine (Agilent Technologies). The PCR program of three segments was as follows: segment 1 was 10 min at 95°C; segment 2 was 40 cycles of 30 s at 95°C, 60 s at 55°C, and 60 s at 72°C; and segment 3 was 60 s at 95°C, 30 s at 55°C, and 30 s at 95°C.

Elongation factor 1 α (*Ef1α*) was used as a reference gene. Primer sequences for *EF1α*, *HAK6*, *SOS1*, and *HKT1.2* were obtained from a previous study by Böhm *et al*. (2018). Primers for *NHX1* were based on the AUR62000862 accession from the ChenopodiumDB database (Jarvis *et al*., 2017) and were as follows: forward, 5′‐TTGGGGTGGTGGGTACATTA‐3′ and reverse, 5′‐ACGCTGAGCTGTGTCAAACC‐3′. All primer sets were run in quadruplets, and the numbers presented in Fig. 3f–i are based on copy numbers of the gene of interest per 10 000 copies of *EF1α*.

### Microscopy analysis

For detailed micrographs and microscopy analysis, a stereo fluorescence microscope (M205 FA; Leica, Wetzlar, Germany) was used. Individual EBCs were additionally observed with a stereomicroscope (EZ4; Leica) over a period of 2 wk to monitor changes in turgidity.

### Statistical analysis

Analysis was performed using Excel 2016 (Microsoft, Redmond, WA, USA) and graphs were plotted using Prism 9 (GraphPad Software, San Diego, CA, USA). Statistical differences were calculated either by Student's *t*‐test (for pairwise comparisons) or one‐way analysis of variance (ANOVA) followed by Tukey's test or Šídák's multiple comparison test (for multiple‐group comparison).

## Results

### The *rebc‐3 rebc‐like1‐1* mutations completely suppress bladder development

To investigate the role of EBCs in salt tolerance using an unbiased approach, we searched for EBC‐free mutants. Ethyl methanesulfonate‐mutagenized plants (*M*_2_ generation) of quinoa cv 5206 were grown under field conditions and screened for individuals with altered EBC densities. One individual plant was identified that showed no detectable EBCs. The mutant plant in the field was likely to have been cross‐pollinated by neighbouring plants, which explains why some of its progeny (*M*_3_ generation) did develop EBCs, while most had various degrees of reduced EBC densities. Generally, in the *M*_3_ generation, we detected two different phenotypes: leaves without EBCs (EBC‐free) and leaves with normal EBC distribution (WT‐like). After self‐pollination under glasshouse conditions, individual EBCs could be spotted on the emerging leaves and flower buds of some *M*_4_ EBC‐free‐leaf plants, but not on others. One plant completely lacking EBCs not only on the surface of its leaves but also on its inflorescences and flower buds was selfed, and its progeny (*M*_5_ generation) were used for further experiments (see Fig. S1a for a brief illustration of how the investigated plants were identified via their leaf phenotype from the *M*_2_ to *M*_5_ generations).

**Fig. 1:**
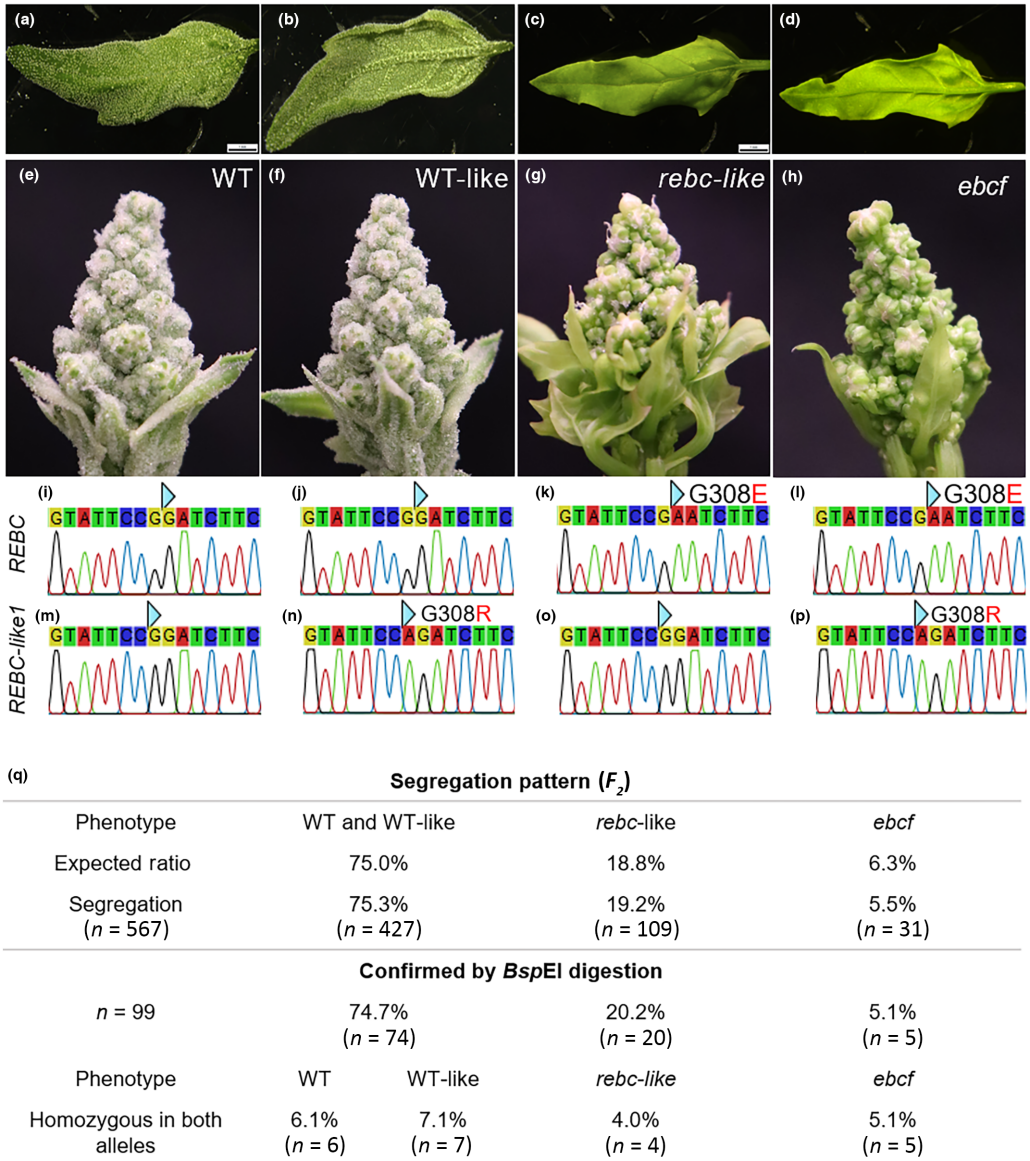
Double mutants of both REBC and REBC‐like1 show a complete loss of epidermal bladder cells (EBCs). Wild‐type (WT) and WT‐like *Chenopodium quinoa* plants possessing the WT allele of *REBC* (i, j) had leaves with high EBC densities on both the adaxial (a) and abaxial (b) sides, as well as on inflorescences (e, f). The *rebc‐like1‐1* allele (G to A, resulting in G308 to R) present in WT‐like plants (m, n) did not alter the abundance or distribution of EBCs. When *REBC* was affected (*rebc‐3*, G to A, resulting in G308 to E; (k), (l)), no EBCs were present on the leaves (c, d); however, some EBCs (epidermal bladder cells) were present on the inflorescences when plants possessed the WT allele of *REBC‐like1* (g, o). Double mutants were completely devoid of EBCs (*epidermal bladder cell‐free* (*ebcf)*; (h), (p)). The blue triangle indicates the position of mutation. Bars, 1 mm. *rebc‐3* and *rebc‐like1‐1* showed a segregation ratio in the F_2_ generation, suggesting that both segregate as recessive alleles in a Mendelian manner, which was confirmed by *Bsp*EI digestion (q).

Although the aim of this work was to investigate the role of EBCs in salt tolerance, we wanted to determine why *ebcf* plants completely lacked EBCs, whereas previously characterized *rebc* mutants still form EBCs, albeit at a reduced density (Imamura *et al*., 2020). As EBC development has previously been shown to involve *REBC* (Imamura *et al*., 2020), we first focused on identifying mutations that could have been induced in *REBC* via EMS mutagenesis. For this, we crossed the *ebcf* mutant with a WT plant and sequenced *REBC* in the *F*_3_ progeny. Sanger sequencing of *REBC* identified a homozygous G923A point mutation in all plants lacking EBCs on their leaves (Fig. 1c,d,k,l), while plants with a WT copy of *REBC* did not show an altered EBC pattern (Fig. 1a,b,i,j). This point mutation was predicted to cause a Gly308Glu substitution in which a buried uncharged Gly residue is replaced with a negatively charged Glu residue (Fig. 1k,l). This mutant allele was different from the previously identified mutant alleles *rebc1* and *rebc2* (Imamura *et al*., 2020) and was named *rebc‐3*.

We also screened for mutations in *REBC‐like1* (*AUR62009292*), a homologue of *REBC* (Imamura *et al*., 2020). In this gene, we detected a G922A substitution, which was predicted to cause a Gly308Arg transition in REBC‐like1, which would replace Gly308 with a positively charged Arg residue (Fig. 1p). This mutant allele was named *rebc‐like1‐1*. Thus, the *rebc‐3* and *rebc‐like1‐1* mutant alleles were predicted to result in a substitution at the same position in both encoded proteins, resulting in the change of a glycine to a negatively and a positively charged residue, respectively (Fig. 1).

Interestingly, the mutation in *REBC‐like1* alone did not have any effect on EBC patterning (Fig. 1a,b,f,j,n). We called this plant WT‐like. However, when the *REBC‐like1* mutation occurred together with a mutation in *REBC*, the plants completely lacked EBCs, not only on the leaves, but also on the inflorescences (Fig. 1c,d,h,l,p). We refer to these mutant plants as *epidermal bladder cell‐free* (*ebcf*). Plants with EBC‐free leaves carrying only the REBC mutation still showed EBCs on the inflorescences (Fig. 1c,d,g,k,o). In all cases, heterozygous mutations were not sufficient to cause the *ebcf* mutant phenotype.

We screened the *F*_*3*_ population and found that, of 567 plants, 353 (75.4%) had a normal EBC distribution, while 89 (19.0%) plants had reduced EBC densities, and 26 (5.6%) had no EBCs at all, fitting the expected segregation for our findings (Fig. 1q). As both point mutations lay on the restriction site (5′‐TCCGGA‐3′) recognized by the *Bsp*EI restriction enzyme, we used restriction enzyme digestion to identify both *rebc‐3* and *rebc‐like1‐1* (Fig. S1b,c). By genotyping 99 of the 567 plants through *Bsp*EI digestion, we confirmed that both *rebc‐3* and *rebc‐like1‐1* segregated as recessive alleles in a Mendelian manner and that the combined homozygosity of both resulted in the *ebcf* phenotype (Fig. 1q).

### The complete loss of epidermal bladder cells does not correlate with a salt stress response penalty

Despite lacking EBCs, *ebcf* plants appeared vital with respect to physiological parameters assessed under growth chamber conditions, such as transpiration, assimilation, leaf temperature, and *F*_v_ */F*_m_, regardless of whether or not the plants were exposed to salinity (50 and 400 mM) (Fig. S4a–d).

**Fig. 2:**
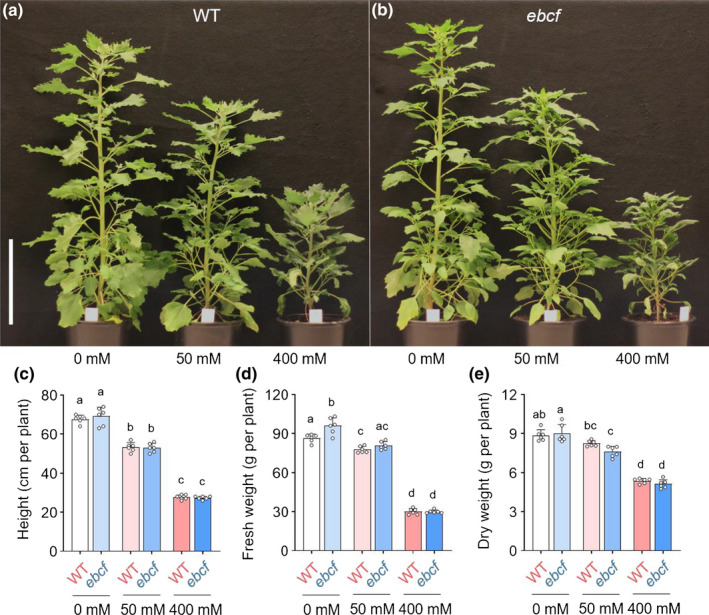
The salt tolerance of a mutant lacking epidermal bladder cells (EBCs) (*ebcf; epidermal bladder cell free*) is unchanged compared with the wild‐type (WT) in *Chenopodium quinoa*. (a, b) Irrigation with 50 and 400 mM salt (NaCl) for 35 d decreased the growth of WT (a) and *ebcf* (b) plants (56 d after sowing (DAS)), but all plants remained viable. Bar, 20 cm. (c–e) Recordings of height (c), fresh weight (d), and dry weight (e) show no differences in growth between the WT and *ebcf* regardless of the treatment used. Values are mean ± SD (*n* = 6 plants). Different letters indicate significant differences (*P* < 0.05) from a one‐way analysis of variance (ANOVA) followed by Tukey's multiple comparison test. Individual values are presented as open circles.

To determine the possible role of EBCs in the salt tolerance of quinoa, we grew the *ebcf* mutant under control (0 mM NaCl), mild (50 mM NaCl), and severe (400 mM NaCl) salt stress conditions. In all cases and regardless of the salt treatment, *ebcf* plants showed similar growth to the WT plants (Fig. 2a–e). Salt tolerance indexes (STIs, reduction in FW compared to control) were identical for the WT and *ebcf* plants (Fig. S4e). Therefore, no difference in tolerance to salinity was detected between WT and *ebcf* mutant plants.

**Fig. 3:**
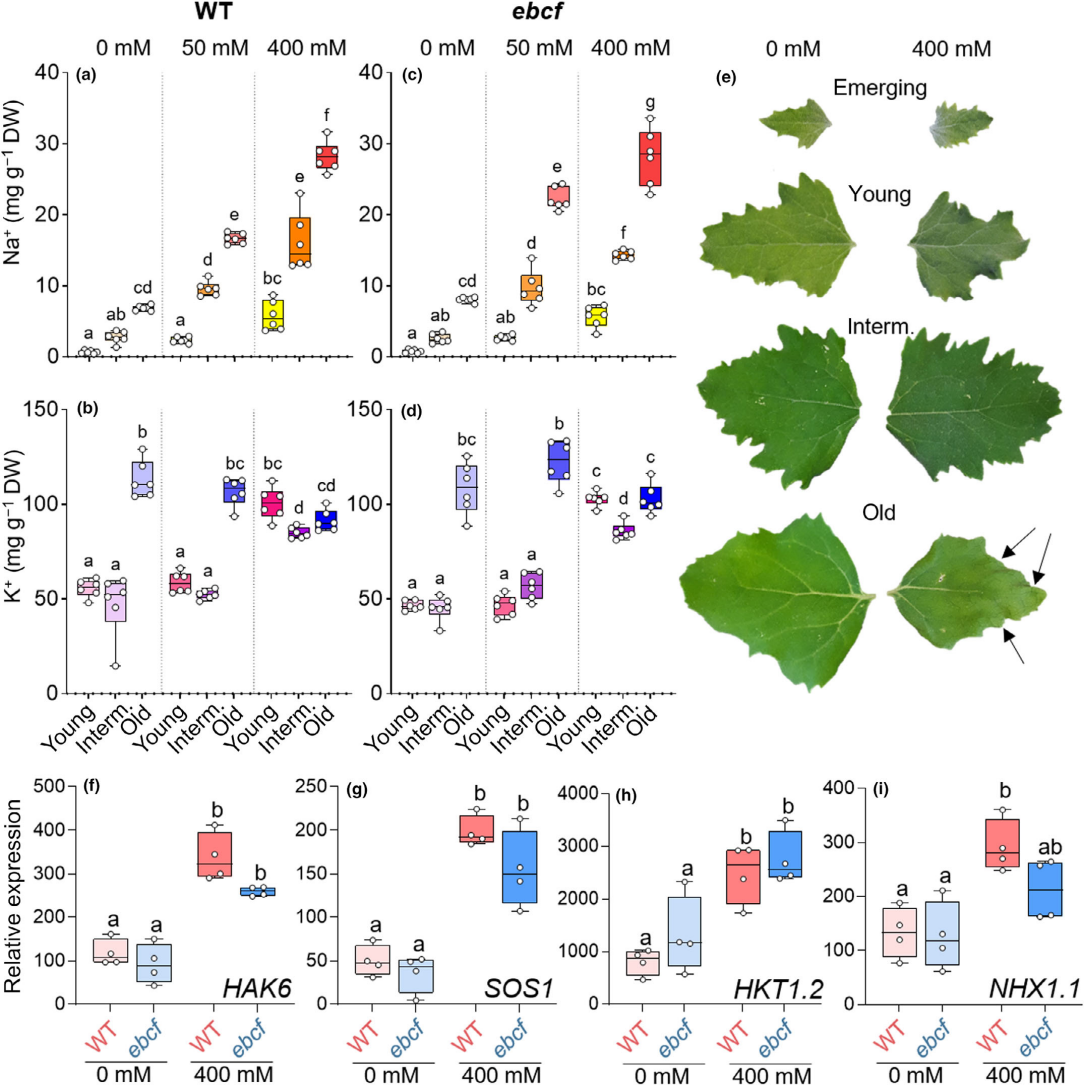
Sodium (Na^+^) and potassium (K^+^) uptake is not altered between the WT (wild‐type) and *ebcf* (*epidermal bladder cell free*) in *Chenopodium quinoa*. Both Na^+^ and K^+^ concentrations increase following irrigation with increasing salt (NaCl) concentrations (4 wk of irrigation with 0, 50, or 400 mM NaCl). While Na^+^ was loaded into old and intermediate‐aged (interm.) leaves regardless of treatment (a), K^+^ predominantly accumulated in old leaves only during control and mild NaCl treatment, but was found in high concentrations under 400 mM NaCl treatment (b). In each case, leaves of *ebcf* accumulated similar amounts of Na^+^ (c) and K^+^ (d) to the WT. In addition, genes encoding the K^+^ transporter HAK6 (f), as well as the Na^+^ transporters SOS1 (g), HKT1.2 (h), and NHX1.1 (i), were not differentially expressed between the mutant and WT plants, under either control conditions or after 400 mM NaCl irrigation (determined by real‐time quantitative polymerase chain reaction (RT‐qPCR)). The boxes extend from the 25^th^ to the 75^th^ percentiles, with whiskers ranging from minimum to maximum values. The horizontal lines in the boxes indicate the medians (*n* = 6 for ion measurements; *n* = 4 for expression analysis). Different letters indicate significant differences (*P* < 0.05) from a one‐way analysis of variance (ANOVA) followed by Tukey's multiple comparison test. When WT plants were irrigated with 400 mM NaCl, only the old leaves suffered from ion toxicity and started to die, beginning from the edges, as indicated by arrows in (e). Individual values are presented as open circles.

Following salt treatment, young, intermediate, and old leaves of *ebcf* plants were analysed for their Na^+^, K^+^, and Cl^−^ content. Compared to the WT, the Na^+^, K^+^, and Cl^−^ content of leaf tissues was not altered by any treatment or at any stage of leaf development (Figs 3a–d, S5a,b; Table S1). Thus, the WT and *ebcf* plants accumulated the same amount of Na^+^ under our experimental conditions. We found this observation to be in line with the expression patterns of genes encoding known K^+^ (*HAK6*) and Na^+^ (*SOS1*, *HKT1.2*, and *NHX1.1*) transporters in quinoa: the relative expression of all of these genes was increased in salt‐treated plants, whereas no differences in expression were observed between the WT and *ebcf* (Fig. 3f–i).

### Mechanical removal of epidermal bladder cells did not increase susceptibility in cultivars with contrasting salt tolerance

To test whether EBCs might function in salt tolerance in other cultivars, we obtained plants devoid of EBCs by mechanically removing the EBCs by brushing. For this purpose, we selected a variety of quinoa cultivars that differed with respect to EBC density, ability to accumulate Na^+^ in leaves and apparent salt tolerance. Thus, in addition to the Titicaca cultivar (cv 5206), the selection included five cultivars that had previously been classified as tolerant or sensitive according to Kiani‐Pouya *et al*. (2019): cv 144 (sensitive), cv 149 (tolerant), cv 189 (tolerant), cv 197 (sensitive), and cv 350 (sensitive) (see Fig. S6m–x for images of these plants). These cultivars differed in EBC densities, with EBCs contributing from 14% (cv 189) to 23% (cv 144) of the total biomass of young leaves (Fig. S7f–j), and showed high variation in Na^+^ accumulation per leaf when NaCl was supplied in the irrigation water (Fig. S7a–e).

Each of the six selected cultivars was divided into two groups, a control group and a group subjected to brushing. After 5 wk of 400 mM NaCl treatment, no cultivar showed reduced growth when brushed compared to its nonbrushed counterpart (Fig. S6a–f). Surprisingly, this was not the case for all cultivars under control conditions (0 mM NaCl): cv 189 showed increased growth when its EBCs were removed, whereas cv 350 showed decreased growth. In cv 189, the increase in growth under control conditions led to a decreased relative salt tolerance, as measured by relative fresh weight compared to control plants (Fig. S6i); in cv 350, the decreased growth under control conditions was associated with an increased relative salt tolerance (Fig. S6k). The cv 5206 plants did not show any differences when grown under control conditions or when treated with salt (Fig. S6f,i). In conclusion, we observed a lack of correlation between the presence of EBCs and salt tolerance in the quinoa cultivars tested.

### Sodium primarily accumulates in old leaves in plants under salt stress

We next compared the Na^+^ concentrations of young, intermediate, and old leaves. After a prolonged period of salt stress (50 and 400 mM NaCl), mature leaves (intermediate and old) of quinoa cv 5206 accumulated significantly more Na^+^ than young leaves (Fig. 3a). The Na^+^ concentration was significantly higher in mature leaves of plants subjected to moderate (50 mM) salt stress than in those of nontreated plants. However, the Na^+^ concentration in young leaves was not significantly altered by moderate salt treatment (Fig. 3a). Potassium concentrations and K^+^ : Na^+^ ratios were unaltered under moderate salt stress conditions, but they doubled in leaves of young and intermediate age when severe salt stress (400 mM) was applied (Figs 3b, S8a). It is of note that K^+^ concentrations remained unchanged in old leaves (Fig. 3b). Old leaves with low K^+^ : Na^+^ ratios underwent progressive necrosis, starting from the outer edges of the leaf (Fig. 3e). In summary, and the majority of Na^+^ taken up under salt stress conditions ended up in old leaves, while K^+^ tended to accumulate in young leaves.

### Epidermal bladder cells do not accumulate enough sodium to act as salt dumps

To determine the distribution of Na^+^, a detailed ion analysis of EBCs was performed. For this purpose, EBCs were gently scraped from both adaxial and abaxial sides of the leaf, resulting in a large quantity of EBCs, which showed no visible signs of damage when checked under the microscope, and an EBC‐free leaf portion (Fig. S3a–d). Weighing leaves before and after scraping revealed that the EBCs accounted for *c*. 36% (w/w) of the total leaf mass of emerging leaves and 10% of the total leaf mass of young leaves in cv 5206 (Fig. S3e,f). The water content of turgid EBCs was around 90%, while the underlying young leaf had a water content of 80% (Fig. S3g; Table S2).

In the EBCs of quinoa cv 5206, the Na^+^ concentration increased rapidly within 24 h of salt application, from 2.5 to 10 mM, but remained below *c*. 60 mM even after prolonged stress (30 d) (Fig. S9a,b). The Na^+^ concentration was significantly lower in emerging than in young leaves of the salt‐treated plants (Fig. S9c). The *in vivo* Na^+^ concentration in EBCs was also significantly lower than in the underlying leaf tissue when based on fresh weight (Fig. S9i). We then tested whether this pattern of Na^+^ distribution extended to other cultivars with varying proportions of EBC biomass and salinity tolerances, including those that were highly tolerant to salt stress (Kiani‐Pouya *et al*., 2019). Irrespective of the cultivar, EBCs did not accumulate Na^+^ to higher concentrations than 20 mM after 5 wk of irrigation with 400 mM NaCl (Fig. S9d).

**Fig. 4:**
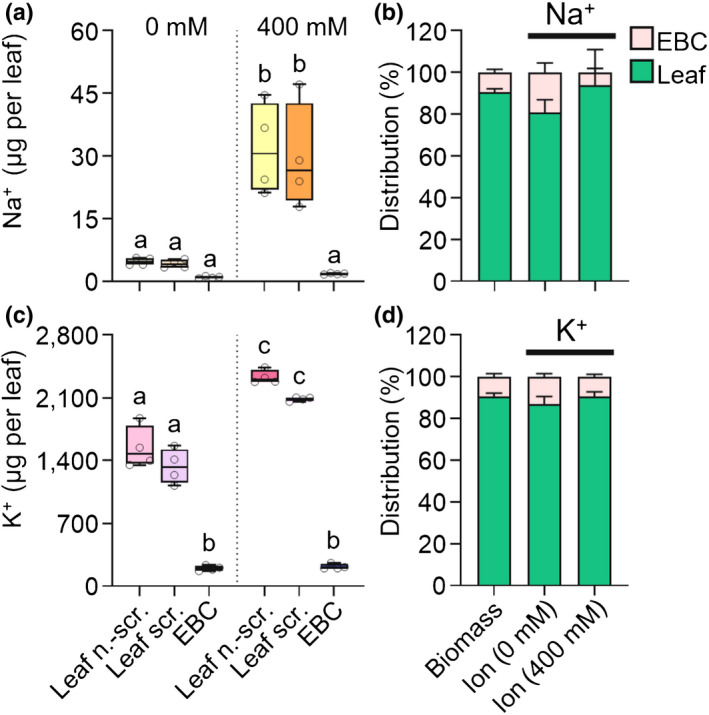
Epidermal bladder cells (EBCs) do not act as sodium (Na^+^) dumps in *Chenopodium quinoa*. (a–d) Ionic analysis showing the distribution of ions within leaves (after standardising leaf weight) by comparing one (unscraped) leaf half with the other leaf half, which was scraped and divided in the two portions: EBCs (epidermal bladder cells) and the underlying leaf. (a) During control conditions, little Na^+^ was measured in either portion; however, Na^+^ increased when treated with 400 mM salt (NaCl), but mainly in the leaf portion, resulting in a distribution‐shift within the leaf. (b) While *c*. 20% of total Na^+^ was found in EBCs during control conditions, only *c*. 5% was found in these cells during salt stress, even though the total EBC‐biomass fraction of the leaf was *c*. 10% in both cases. (c) Total potassium (K^+^) concentrations were much higher in all leaf portions (i.e. nonscraped leaf, scraped leaf and epidermal bladder cells). (d) Epidermal bladder cells did not take up enough K^+^ to remove significant amounts of the total K^+^ from the leaf. Also, the K^+^ distribution in EBCs was less affected by the salt treatment than the Na^+^ distribution was (*c*. 13% under 0 mM NaCl irrigation; *c*. 10% under 400 mM NaCl irrigation). The boxes extend from the 25^th^ to the 75^th^ percentiles, with whiskers ranging from minimum to maximum values. The horizontal lines in the boxes indicate the medians (*n* = 4). Different letters indicate significant differences (*P* < 0.05) from one‐way analysis of variance (ANOVA) followed by Tukey's multiple comparison test. The stacked bars show mean values (*n* = 4) with SD. Individual values are presented as open circles. Leaf n.‐scr., nonscraped leaf; Leaf scr., scraped leaf.

Next, we investigated the fraction of total leaf Na^+^ the EBCs were able to accumulate. For this purpose, a leaf was cut in half longitudinally and the EBCs were scraped from one half, resulting in a leaf half with EBCs (to analyse the total Na^+^ accumulation of a leaf), a leaf half with no EBCs, and a quantity of EBCs that had been removed from the latter half. In this way, the distribution of Na^+^ within the leaf was calculated. When the plants were irrigated with NaCl, Na^+^ increased in leaves but only marginally in EBCs (Fig. 4a). When treated with 400 mM NaCl, 31.7 μg Na^+^ accumulated in the nonscraped leaf‐half. More importantly, only 1.8 μg of the Na^+^ was found in the EBCs, leaving 29.5 μg in the underlying leaf (Fig. 4a), which implies that the EBCs were only able to remove a minor fraction (6%) of the total Na^+^ from the leaves (Fig. 4b), even though the biomass fraction of the EBCs was as high as 10% in the analysed leaves. Even though the biomass fraction of EBCs was higher (14–23%) in all other cultivars analysed (Fig. S7f–j), EBCs were not able to take up notable amounts of Na^+^ from the leaves, with the exception of cv 149, which has been classified as salt tolerant (Fig. S7a–e).

Inconsistent with the salt dump hypothesis, cv 350, which showed improved salt tolerance when brushed (Fig. S6k), accumulated Na^+^ in EBCs to the highest extent (24%) (Fig. S7j). Furthermore, in cv 189, a cultivar that showed reduced salt tolerance when brushed (Fig. S6i), the fraction of Na^+^ in EBCs was the lowest in all observed cultivars (3%) (Fig. S7h). Taken together, these results indicate that directing Na^+^ to EBCs did not make a much of a contribution to salt tolerance.

### Epidermal bladder cells accumulate extra‐high quantities of potassium

In contrast to Na^+^, K^+^ concentrations in EBCs increased markedly in response to salt stress. When grown under control conditions, the K^+^ concentration of EBCs was around 0.5 M and increased to *c*. 1.5 M in severely salt‐stressed plants (Fig. S9e–h). It should be noted that the K^+^ concentration was found to be higher in EBCs than in the underlying leaf regardless of irrigation management (Fig. S9j). The relative distribution of K^+^ into EBCs was consistent across all tested cultivars (Figs 4c,d, S7p–t). However, in cultivars with a higher proportion of EBC biomass (Fig. S7p–t), EBCs did reduce the total K^+^ concentration in the leaf under both control and salt conditions (Fig. S7k–o). Thus, K^+^, rather than Na^+^, was the dominant cation in EBCs of the cultivars tested, both before and after a prolonged period of salt stress. The unequal distribution of K^+^ and Na^+^ in EBCs therefore resulted in higher K^+^ : Na^+^ ratios in these cells relative to the underlying leaf (Fig. S9k).

### Potassium is recycled from old epidermal bladder cells

**Fig. 5:**
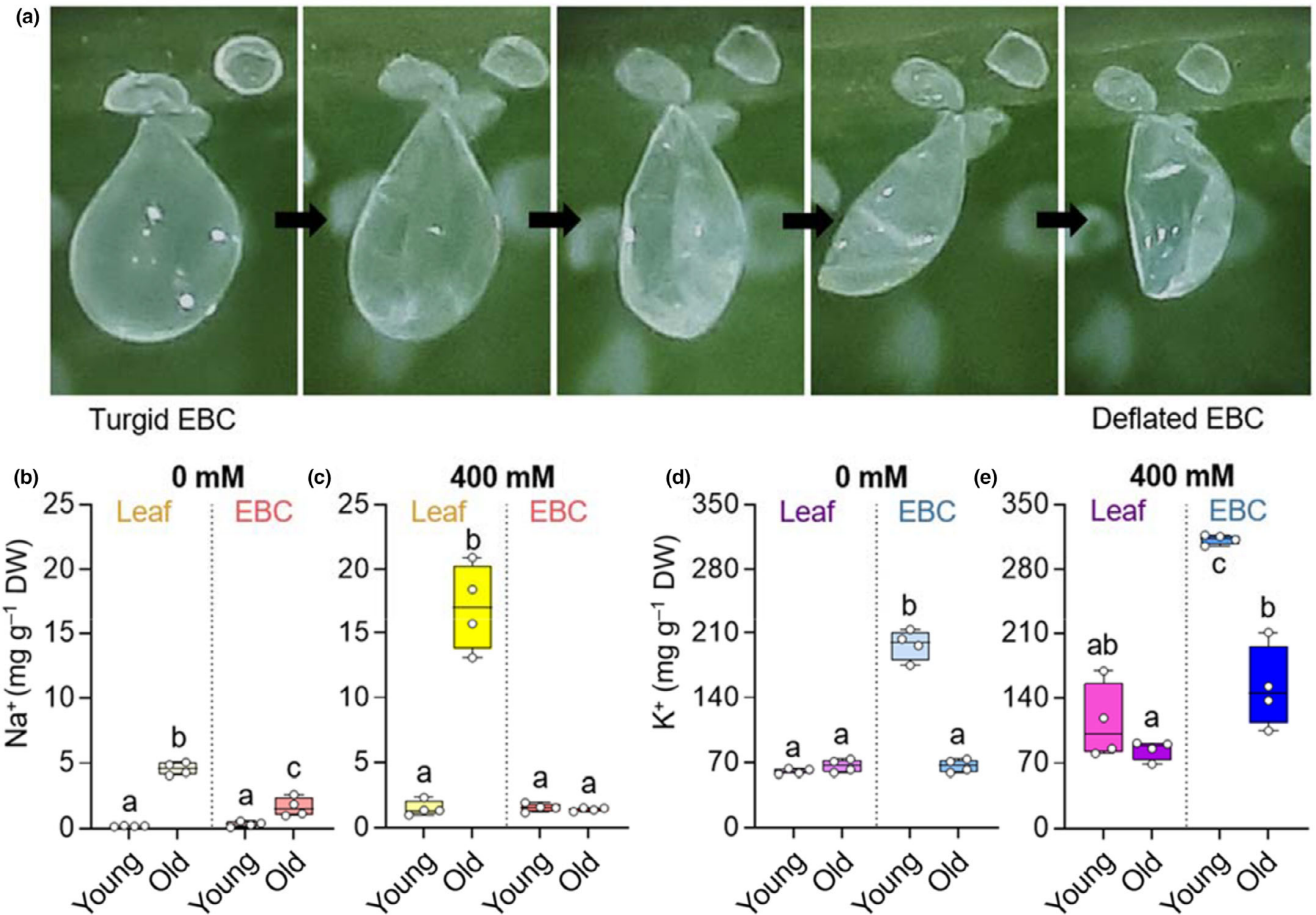
Potassium (K^+^) is recycled from old epidermal bladder cells (EBCs) in *Chenopodium quinoa*. While the low amount of sodium (Na^+^) present in old epidermal bladder cells (EBCs) remains in these cells (b, c), the K^+^ is taken up into the leaf again from these cells during maturity (d, e). This initiates a gradual deflation of EBCs over a period of several days (a). The boxes extend from the 25^th^ to the 75^th^ percentiles, with whiskers ranging from minimum to maximum values. The horizontal lines inside the boxes indicate the medians (*n* = 4). Different letters indicate significant differences (*P* < 0.05) from one‐way analysis of variance (ANOVA) followed by Tukey's multiple comparison test. Individual values are presented as open circles.

Epidermal bladder cells are mostly present on young leaves (Fig. S10a). Old leaves possessed far fewer EBCs, which were flattened and had lost their turgor (Fig. S10b). Instead of a tattered structure, the ‘flat’ EBCs exhibited a bowl‐like structure, like a deflated balloon (Fig. S10c–g). We observed the process of EBC shrinkage in individual EBCs of leaves of intermediate age and established that the EBCs gradually lose turgidity rather than integrity as the leaves mature (Fig. 5a). When comparing the ion concentrations in turgid EBCs present on young leaves to those in deflated EBCs (30% water content, Fig. S3g) present on old leaves, we found that around half of the K^+^ was recycled under both salt stress and control conditions (Fig. 5d,e; Table S3). At the same time, Na^+^ remained in the EBCs (Fig. 5a,b; Table S3). In this way, essential K^+^ is not lost when old leaves are shed.

### Chloride concentrations increase strongly in the underlying leaf fraction and epidermal bladder cell‐fraction under salt stress

Like K^+^, Cl^−^ accumulated to a greater extent in EBCs than in other leaf tissues, and concentrations increased as the severity of the salt stress treatment increased (Fig. S5c). Under control conditions, the Cl^−^ concentration in EBCs was as low as 10 mM and increased up to 800 mM following treatment with 400 mM NaCl (Fig. S5d–f). As observed for Na^+^, overall Cl^−^ concentrations increased in all parts of the plant when subjected to salt stress. However, in contrast to Na^+^, Cl^−^ concentrations were similar in leaves of all ages (Fig. S5a,b) and were much higher than Na^+^ concentrations (Fig. 3a).

## Discussion

In this work, we have shown that an *ebcf* mutant, which completely lacks EBCs, is as tolerant to salt stress as the WT under identical experimental conditions. Furthermore, we have demonstrated that EBCs only accommodate a negligible fraction of the total Na^+^ in leaves and, in response to salt stress, selectively take up K^+^ and, to only a minor degree, Na^+^. Potassium is recycled when leaves mature and Na^+^ is expelled from the plant when old leaves are shed.

### A mutant lacking epidermal bladder cells does not lose salt tolerance

The *ebcf* mutant did not differ in its salt tolerance response compared to WT plants. In this respect, our results deviate from those of Kiani‐Pouya *et al*. (2017), who found that the mechanical removal of EBCs in quinoa by brushing led to increased salt sensitivity. However, removal of EBCs from WT leaves by brushing is an invasive technique and reduces the fitness of the mechanically stressed plants. As K^+^ is removed when EBCs are brushed off (Fig. S7k–t), brushing of EBCs could lead to K^+^ deficiencies, as can be seen in the salt‐treated brushed plants in Kiani‐Pouya *et al*. (2017), which contained only *c*. 60% of the amount of K^+^ observed in nonbrushed plants. As K^+^ plays an important role in salt‐stress tolerance, the K^+^ deficiency caused by the brushing, rather than the loss of Na^+^ storage capacity, could have led to the reduced fitness when salt treated. No differences in either Na^+^ or K^+^ accumulation were observed between WT and *ebcf* (Fig. 3a–d) plants in the naturally EBC‐free mutant experiments, which could be performed without the application of mechanical stress.

Similar to the approach employed here, Imamura *et al*. (2020) employed EMS mutagenesis to identify two *rebc* mutants of quinoa with a reduced number of EBCs. The *rebc* mutants were slightly more sensitive to salt than the WT plants and displayed damaged shoot apices when exposed to abiotic stress conditions such as wind and UV (Imamura *et al*., 2020). We did not observe a similar phenotype in *ebcf* mutant plants (Fig. S4f). In addition, *ebcf* mutants did not exhibit changes in overall fitness (Figs 2, S4), nor was the overall salt uptake into the shoot affected (Figs 3a–d, S5a,b) or the expression of genes encoding K^+^ and Na^+^ transporters altered (Fig. 3f–i). These observations suggest that the *rebc* mutants described by Imamura *et al*. (2020) suffered from reduced general fitness, possibly resulting from specific growth conditions or genetic alterations in one or more additional genes that compromise the plant's response to external stresses. The *ebcf* mutant described in this work did not show any reduction in fitness under the growth conditions tested, including salt stress.

### Epidermal bladder cells preferentially accumulate K^+^, while Na^+^ is underrepresented in epidermal bladder cells

Orsini *et al*. (2011) had already questioned whether the volume of EBCs was sufficient to play an important role in salt tolerance of quinoa, as did the recent publications by Roman *et al*. (2020) and Otterbach *et al*. (2021). If EBCs were to serve as Na^+^ dumps, they would need to be able to store sizable amounts of Na^+^ to reduce its accumulation in the leaf mesophyll and to protect the rest of the leaf from Na^+^ overload. Here, we show that quinoa EBCs contained only low concentrations of Na^+^ (*c*. 5 mM) under control conditions and, even though the concentration increased due to salt treatment, it remained low under all conditions (Fig. S9a–d) – far from the proposed 0.85 M (Kiani‐Pouya *et al*., 2017). Not only were EBCs unable to remove significant amounts of Na^+^ from the leaf (Figs 4a, S7a–e), but based on fresh weight concentrations of Na^+^ were actually lower in EBCs than in the underlying young leaf tissues (Fig. S9i).

In this study, we established that concentrations of K^+^ increased sharply in EBCs in response to salt stress. Under control conditions, K^+^ concentrations were already as high as 0.5 M and increased up to 1.7 M in the EBCs of salt‐stressed plants (Fig. S9e–h). Regardless of the severity of the salt stress, the K^+^ concentration of EBCs far exceeded that of the leaf (Figs 5d,e, S9j). The concentration of Cl^−^ was also high in EBCs under salt stress, and this ion most likely acts as a ‘cheap’ anion to prevent charge imbalances following the strong increase in K^+^ concentration. This behaviour is in line with the results of Böhm *et al*. (2018) where relative transcription levels of the K^+^ transporter gene *CqHAK5* and the Cl^−^ transporter gene *CqClC‐c* were found to be higher in EBCs than in the rest of the leaf. By contrast, Na^+^ transporter genes such as *CqSOS1* or *CqHKT1.2*, which according to the salt‐dump hypothesis were expected to drive Na^+^ into the EBCs, did not show a preferential expression in these cells. In emerging leaves, we found EBCs to contribute *c*. 35% to the total leaf biomass, and in young leaves, *c*. 10% (Fig. S3e,f). Similar relative expressions mean that the majority of *CqSOS1* and *CqHKT1.2* transcripts are found in the underlying leaf fraction, which would explain why we could not detect relevant differences in the expression of these two genes between WT and *ebcf* plants (Fig. 3g,h). This indicates that EBCs are not specifically equipped to act as salt dumps, but rather accumulate K^+^ followed by water to provide a proper aqueous reservoir for the storage of solutes.

Each EBC complex consists of a leaf epidermal cell, a stalk cell, and the bladder. Under salt stress, potassium (K^+^), chloride (Cl^−^), sodium (Na^+^) to a minor degree, and various other metabolites are shuttled from the leaf lamina to the bladders. Via RNA sequencing and transport of the stalk cells Bazihizina *et al*. (2022) recently showed that these transfer cells express genes encoding ion channels and carriers. In a working model, transcellular transport via HKT1, AKT1/HAK and an MFS mediates stalk cell K^+^, Cl^−^ and Na^+^ uptake from the epidermis and SOS1, SKOR1, and SLAH3 ion release towards the bladder. Future studies will have to demonstrate whether bladder K^+^, Cl^−^, Na^+^ and osmotic water accumulation is reversible.

We have furthermore shown that K^+^, water (Figs 5d,e, S3g), and possibly also other metabolites are reabsorbed by maturing leaves as bladders progressively deflate. Thus, EBCs are more likely to shrink than burst when they mature. Potassium, the main cation identified by us as accumulating in EBCs, is an essential nutrient with multiple functions in plant cells, which explains why it is reabsorbed once the EBCs have fulfilled their as‐yet unidentified role.

### Conclusion and future prospects

We have shown that EBCs accommodate only a negligible fraction of the total Na^+^ in leaves and, in response to salt stress, selectively take up K^+^ and not Na^+^ and therefore should not be considered to function as salt dumps. Instead, and in accordance with previous findings (Hariadi *et al*., 2011; Bonales‐Alatorre *et al*., 2013), Na^+^ is primarily directed to old leaves, thereby keeping concentrations low in young leaves. At the same time K^+^ moves in the opposite direction, creating beneficial K^+^ : Na^+^ ratoldios in these young leaves, which are still susceptible to Na^+^ toxicity. To reduce extensive K^+^ loss, K^+^ is recycled from old EBCs when leaves mature and is taken back into the leaf. That EBCs do not play a role in the salt tolerance of quinoa was confirmed with the *ebcf* mutant, which completely lacks EBCs and is as tolerant to salt stress as the WT.

The findings presented here, showing that EBCs have no effect on a plant's salt tolerance under our experimental conditions’, will refocus the research on halophyte salt tolerance mechanisms and redirect breeding efforts addressing future food security. Epidermal bladder cells, with high concentrations of osmolytes, may serve as a pull factor for water, which would allow them to play roles in metabolite storage (Otterbach *et al*., 2021) and protection of the epidermal layer (LoPresti, 2014). For example, oxalic acid, which accumulates in EBCs of both halophytes (Lüttge *et al*., 1978; Jou *et al*., 2007; Otterbach *et al*., 2021) and glycophytes (Steudle *et al*., 1983; Franceschi & Nakata, 2005), is a well‐characterized herbivore deterrent (Franceschi & Nakata, 2005). Potassium transport into EBCs is a means of driving water transport in the same direction by osmosis. Water is allowed to return to the leaf when required, concomitant with reabsorption of K^+^. It remains to be tested whether EBCs can serve as water sinks and sources under drought stress.

## Author contributions

MWM, MDLT, AFN and MP designed the research; MWM, MDLT, AFN, AKB, CW, JTØ and TW performed the experiments; MWM, MDLT, AFN, SS, RH and MP analysed the data; and MWM, MDLT, AFN, SS, RH and MP wrote the first version of the manuscript. All authors contributed to the final version of the manuscript. MWM, MDLT and AFN contributed equally to this work.

## Supporting information


**Fig. S1** Identification of epidermal bladder cell‐related mutants.
**Fig. S2** Classification of leaf positions.
**Fig. S3** Sampling of young leaves.
**Fig. S4** The physiological parameters of *ebcf* and wild‐type plants were identical.
**Fig. S5** Epidermal bladder cells accumulate high concentrations of chloride.
**Fig. S6** Mechanical removal of epidermal bladder cells does not affect growth at 400 mM NaCl in a variety of quinoa cultivars.
**Fig. S7** Epidermal bladder cells (EBCs) are not able to remove relevant amounts of sodium (Na^+^) from the leaf in cultivars with varying tolerance and EBC fractions.
**Fig. S8** High potassium : sodium (K^+^ : Na^+^) ratios were observed in young leaves only during salt stress.
**Fig. S9** Epidermal bladder cells accumulate K^+^ over Na^+^.
**Fig. S10** Microscopy analysis of epidermal bladder cells.

**Table S1** Raw data and additional data related to Fig. 3.
**Table S2** Raw data and additional data related to Fig. 4.
**Table S3** Raw data and additional data related to Fig. 5.
Please note: Wiley Blackwell are not responsible for the content or functionality of any Supporting Information supplied by the authors. Any queries (other than missing material) should be directed to the *New Phytologist* Central Office.

## Data Availability

The data that support the findings of this study are available in the [Link], [Link] which accompanies this article.
