# Supplementary material for: The epidermal bladder cell‐free mutant of the salt‐tolerant quinoa challenges our understanding of halophyte crop salinity tolerance
Source: New Phytol. 2022 Aug 30;236(4):1409–21. doi: 10.1111/nph.18420 (PMC9804403; doi:10.1111/nph.18420)
Supplement: Supplementary file 1 — Fig. S1 Identification of epidermal bladder cell‐related mutants. Fig. S2 Classification of leaf positions. Fig. S3 Sampling of young leaves. Fig. S4 The physiological parameters of ebcf and wild‐type plants were identical. Fig. S5 Epidermal bladder cells accumulate high concentrations of chloride. Fig. S6 Mechanical removal of epidermal bladder cells does not affect growth at 400 mM NaCl in a variety of quinoa cultivars. Fig. S7 Epidermal bladder cells (EBCs) are not able to remove relevant amounts of sodium (Na+) from the leaf in cultivars with varying tolerance and EBC fractions. Fig. S8 High potassium : sodium (K+ : Na+) ratios were observed in young leaves only during salt stress. Fig. S9 Epidermal bladder cells accumulate K+ over Na+. Fig. S10 Microscopy analysis of epidermal bladder cells. [file NPH-236-1409-s001.pdf]

New Phytologist Supporting Information

Article title: **The *epidermal bladder cell-free* mutant of the salt tolerant quinoa challenges our understanding of halophyte crop salinity tolerance**

Authors: Max William Moog, Mai Duy Luu Trinh, Anton Frisgaard Nørrevang, Amalie Kofoed Bendtsen, Cuiwei Wang, Jeppe Thulin Østerberg, Sergey Shabala, Rainer Hedrich, Toni Wendt, Michael Palmgren

Article acceptance date: 27 July 2022

Supplementary Figures

Fig. S1. Identification of epidermal bladder cell-related mutants

Fig. S2. Classification of leaf positions.

Fig. S3. Sampling of young leaves.

Fig. S4. The physiological parameters of *ebcf* and WT plants were identical.

Fig. S5. EBCs accumulate high concentrations of chloride.

Fig. S6. Mechanical removal of EBCs does not affect growth at 400 mM NaCl in a variety of quinoa cultivars.

Fig. S7. EBCs are not able to remove relevant amounts of Na^+ from the leaf in cultivars with varying tolerance and EBC fractions.

Fig. S8. High K^+/Na^+ ratios only in young leaves during salt stress.

Fig. S9. EBCs accumulate K^+ over Na^+.

Fig. S10. Microscopy analysis of EBCs.

Supplementary Tables (See separate file)

Table S1. Raw data and additional data related to Figure 3.

Table S2. Raw data and additional data related to Figure 4.

Table S3. Raw data and additional data related to Figure 5.

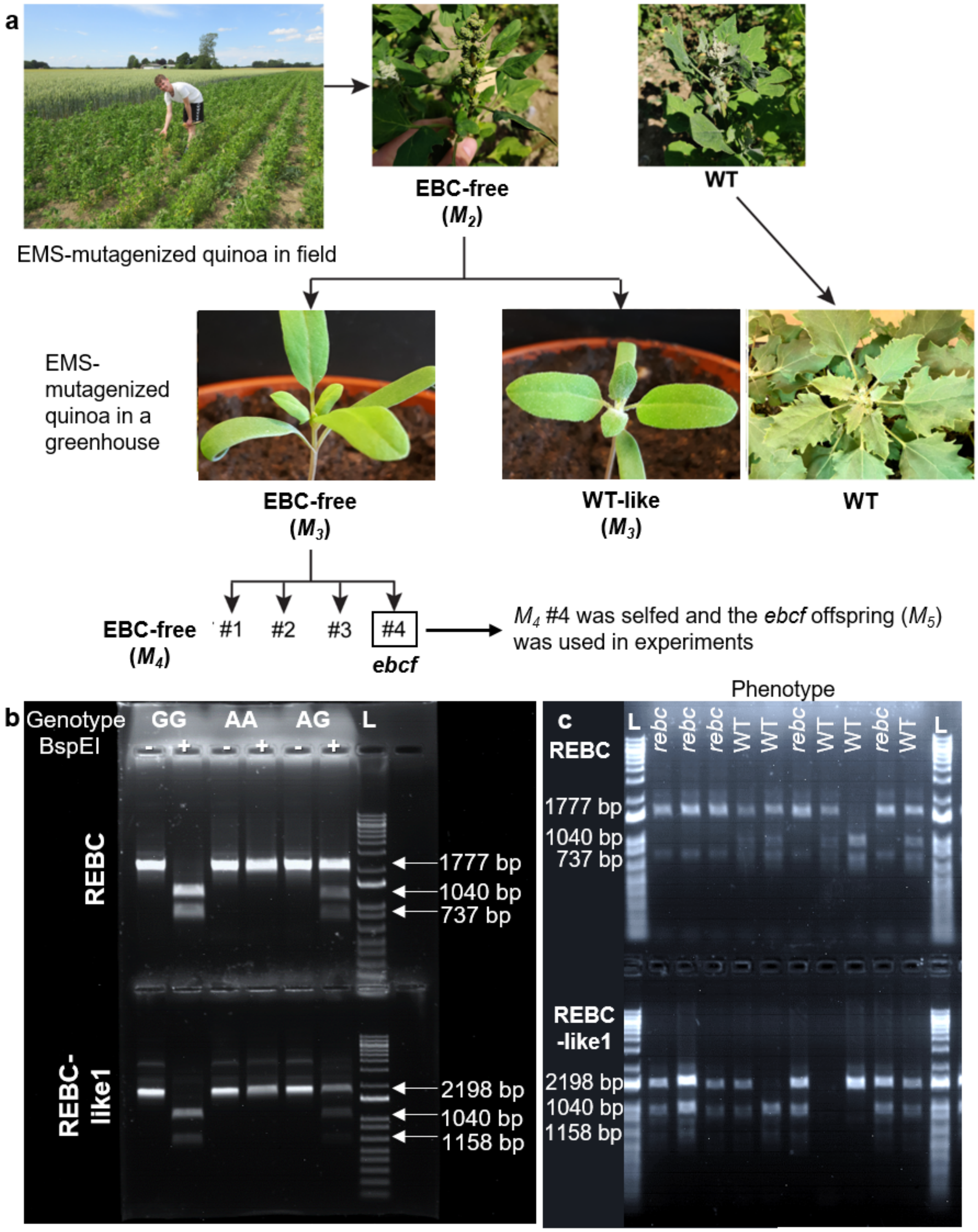

Figure S1. Identification of epidermal bladder cell-related mutants of quinoa. Diagram illustrating identification of plants with EBC-free leaves from M_2 to M_5 progeny. The first EBC-free mutant was identified in the field, and its progeny (M_3 to M_5) were grown and self-pollinated under greenhouse conditions (a). Coincidently, the point mutations in both *rebc-3*

and *rebc-like1-1* lay on a restriction site (5′-TCCGGA-3′) recognized by the *Bsp*EI restriction enzyme. Electrophoresis of amplified *REBC* and *REBC-like1* DNA fragments carrying the WT GG, the mutant AA, or a heterozygous AG genotype with (+) or without (–) *Bsp*EI digestion (**b**). Representative gel after *Bsp*EI digestion of *REBC* and *REBC-like1* DNA fragments obtained from F_2 plants after crossing, used to determine the segregation pattern in Fig. 1q (**c**). L: Invitrogen 1-kb Plus DNA Ladder; arrows indicate the precise length of DNA bands.

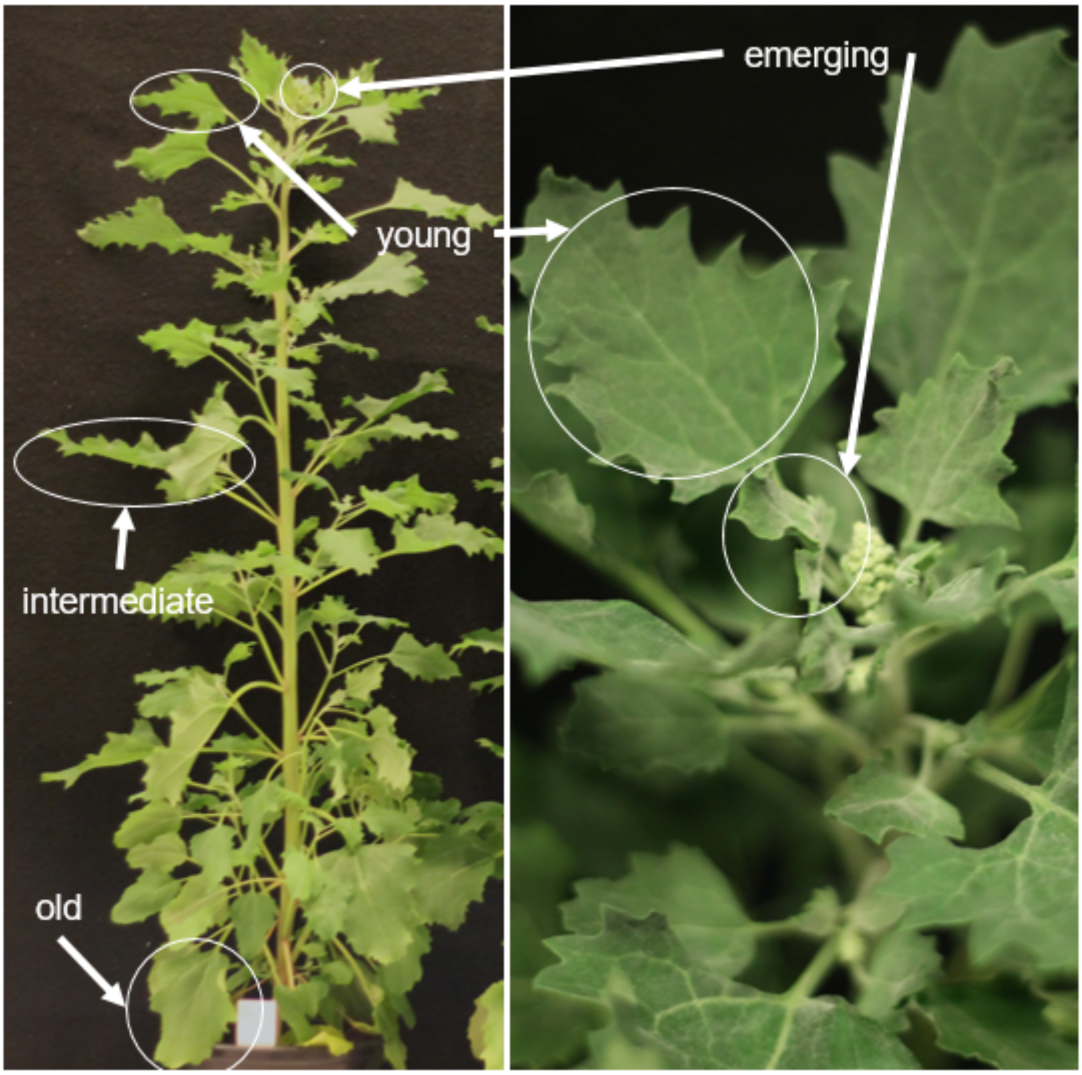

Figure S2. Classification of leaf positions of quinoa. Leaf samples of different ages were classified according to their position on the plant from emerging to young and intermediate to old.

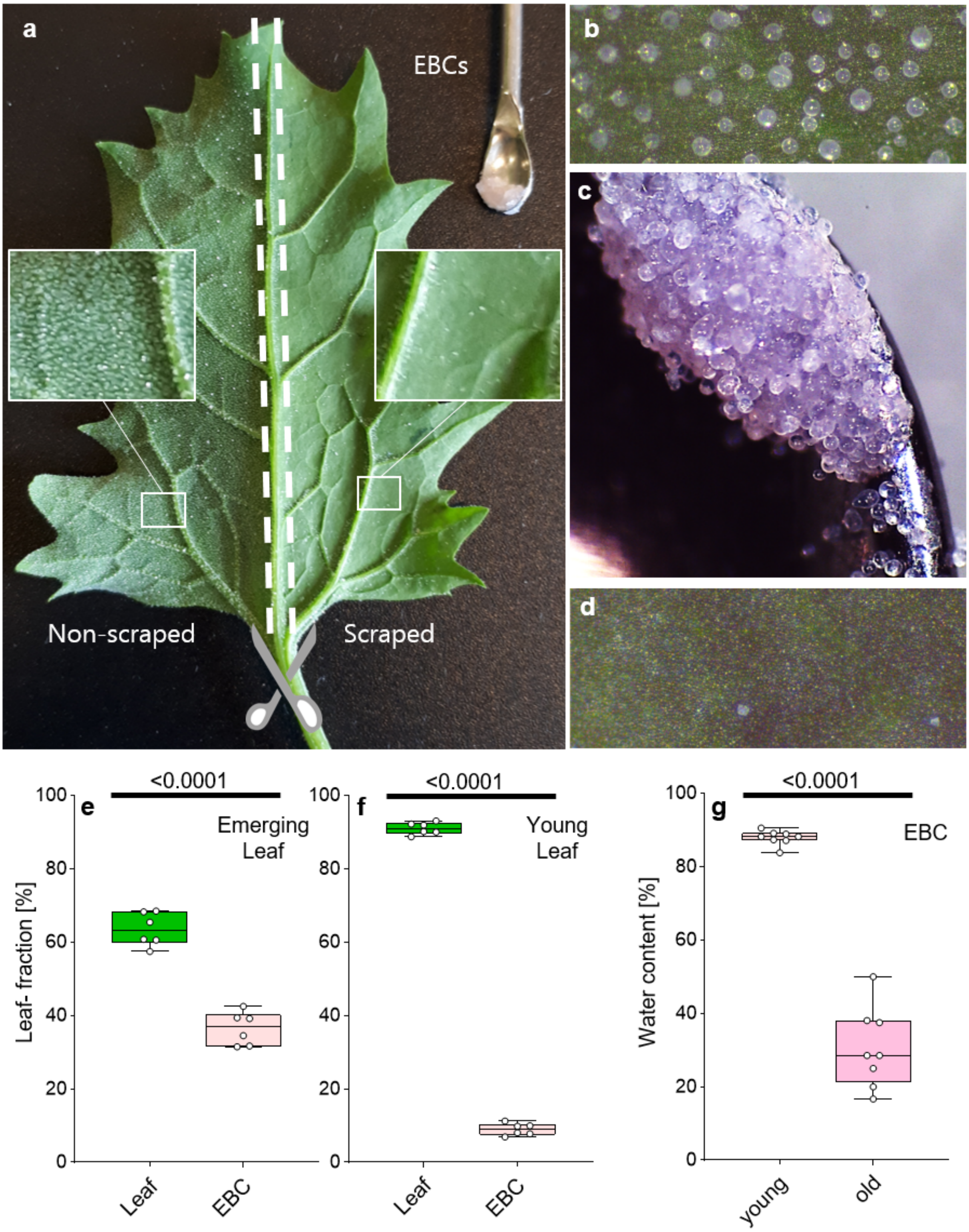

Figure S3. Sampling of young leaves of quinoa. Young leaves with turgid EBCs (epidermal bladder cells) of high density were cut in half longitudinally, excluding the central vascular tissue. While one side remained as it was (Non-scraped), the other half was gently scraped,

resulting in an EBC-free half (Scraped) and an EBC fraction. Insets show enlargements of the boxed regions (**a**). Gentle scraping of leaves covered with EBCs (**b**) resulted in a highly accumulated fraction of intact EBCs (**c**) and almost complete removal of EBCs from the leaf (**d**). EBCs constitute a high percentage of the total leaf biomass in emerging leaves (**e**) but a low percentage in young fully developed leaves (**f**). The water content of turgid young EBCs was around 90% but only 30% in old deflated EBCs (**g**) The boxes extend from the 25th to 75th percentiles, with whiskers ranging from minimum to maximum values. The horizontal lines in the boxes indicate the medians (n = 6). The P-values were calculated by two-tailed Student's t-tests. Individual values are presented as open circles.

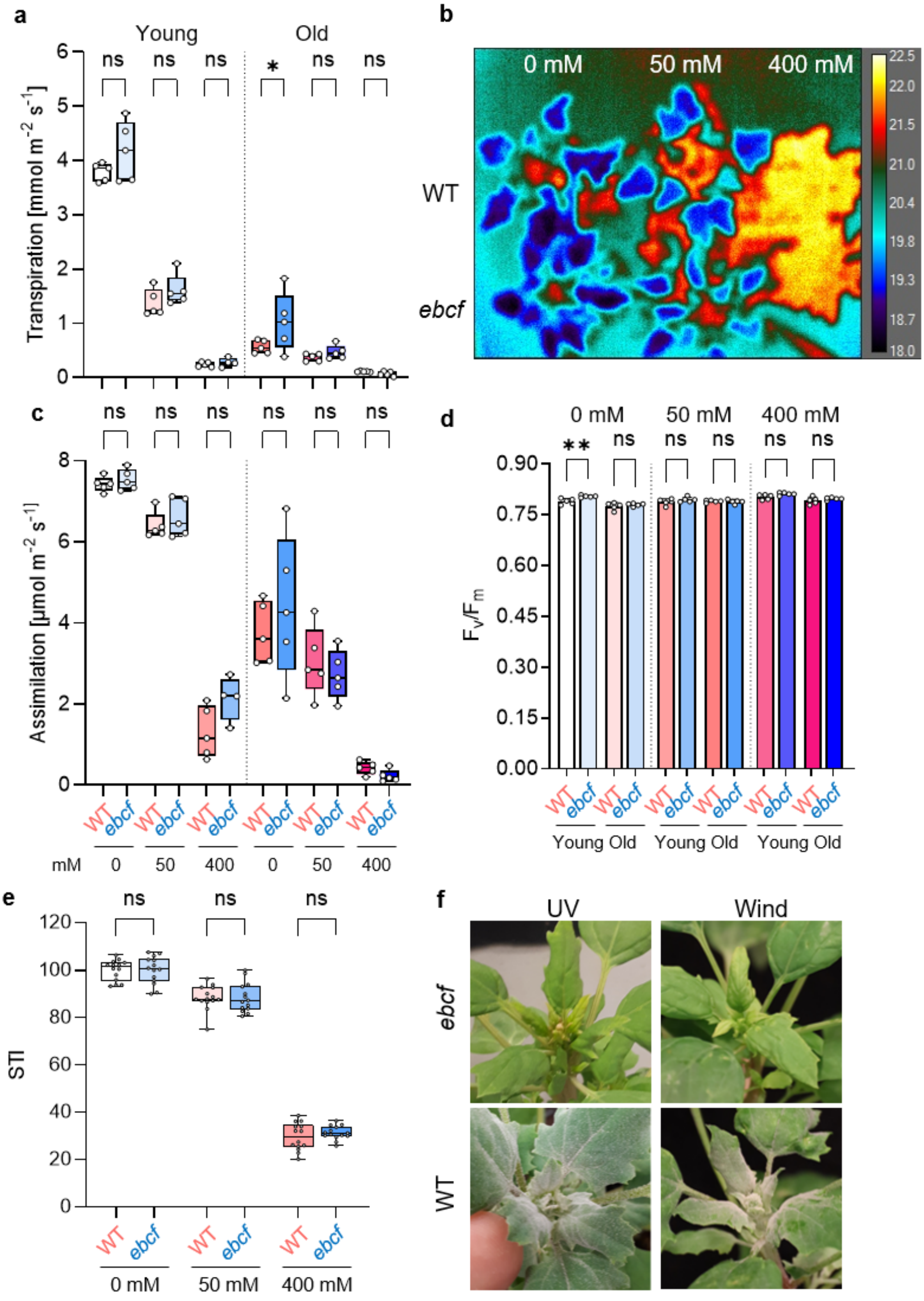
a
Young
Old
Transpiration [mmol m-2 s-1]
ns
*
b
0 mM
50 mM
400 mM
WT
ebcf
22.5
22.0
21.5
21.0
20.4
19.8
19.3
18.7
18.0
c
Assimilation [µmol m-2 s-1]
mM
0
50
400
d
0 mM
50 mM
400 mM
**
Fv/Fm
Young Old
e
STI
0 mM
50 mM
400 mM
f
UV
Wind
ebcf
WT

56

Figure S4. The physiological parameters of *ebcf* and WT quinoa were identical. Transpiration (**a**), thermal image (**b**), assimilation (**c**), and recorded F_v/F_m (**d**) analyses showed no differences between *ebcf* and WT plants grown under various degrees of salinity. The salt tolerance index (STI) represents the relative reduction of FW to control of WT and *ebcf* plants towards salinity from three independent replicates (**e**). The boxes extend from the 25th to 75th percentiles, with whiskers ranging from minimum to maximum values. The lines indicate the medians (n = 6 (**a-c**) and 14 (**e**)). Statistical differences were analysed by one-way ANOVA followed by a Šídák's multiple comparisons test (ns: p value >0.05, *: p value <0.05 and **: p value <0.01). Bar graph (d) with mean values ± s.d. n = 5. Shoot apices of the *ebcf* mutant were not affected by prolonged exposure to UV or wind stress (**f**). Individual values are presented as open circles.

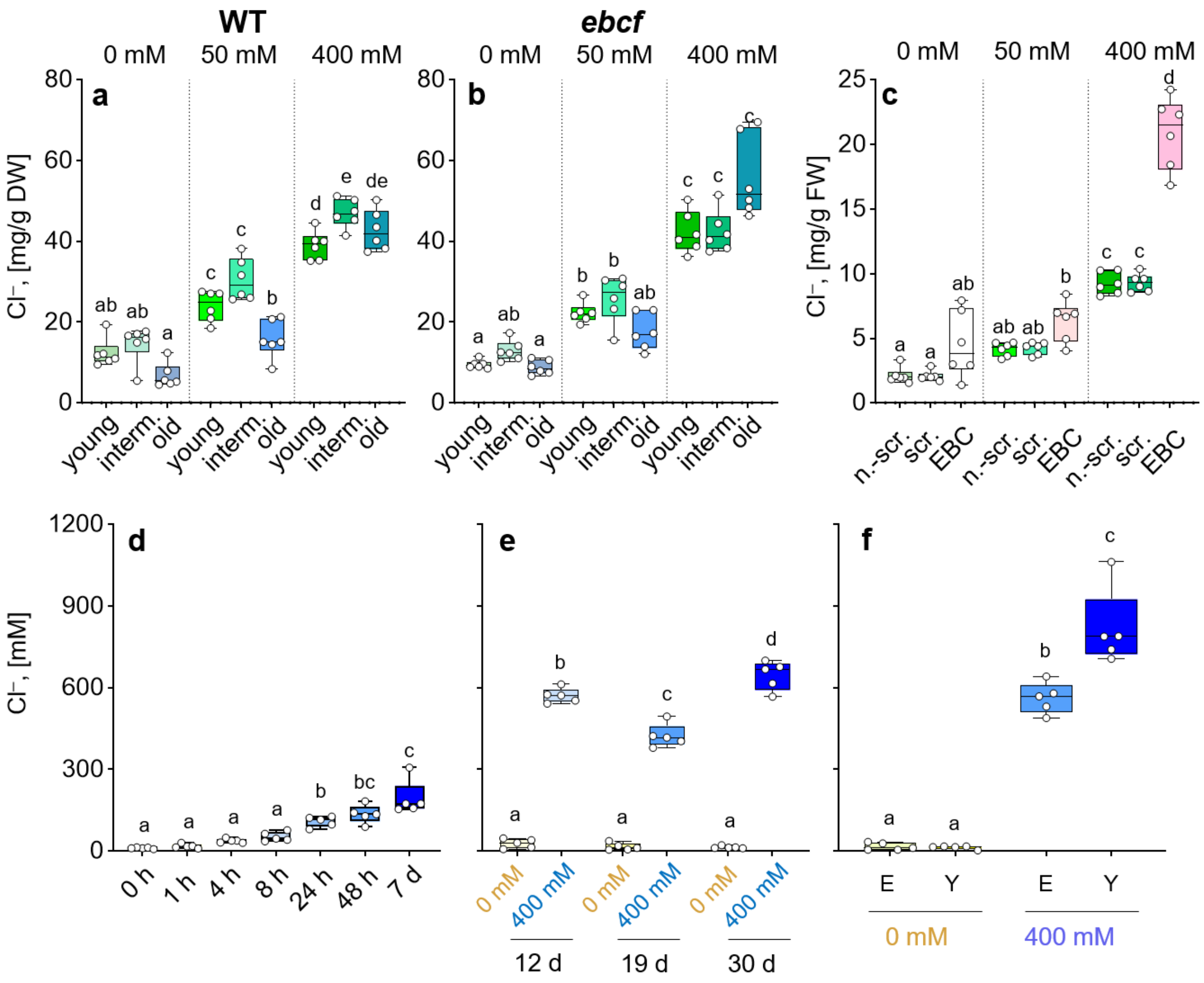

Figure S5. EBCs accumulate high concentrations of chloride. In line with increasing concentrations of NaCl in the irrigation water (0, 50, and 400 mM), Cl^- concentrations increased in both WT quinoa and *ebcf* to the same degree (**a**,**b**). The highest concentrations of Cl^- in the young leaves were found in the EBCs (**c**). While the concentration of Cl^- was as low as 10 mM in EBCs of plants not subjected to NaCl irrigation, it increased up to 800 mM in plants irrigated with 400 mM NaCl (**d**-**f**). The boxes extend from the 25th to 75th percentiles, with whiskers ranging from minimum to maximum values. The horizontal lines in the boxes indicate the medians (n = 6 for a-c, and n = 5 for d-f). Different letters indicate significant differences ($P < 0.05$) from a one-way ANOVA followed by a Tukey's multiple comparison test. Individual values are presented as open circles.

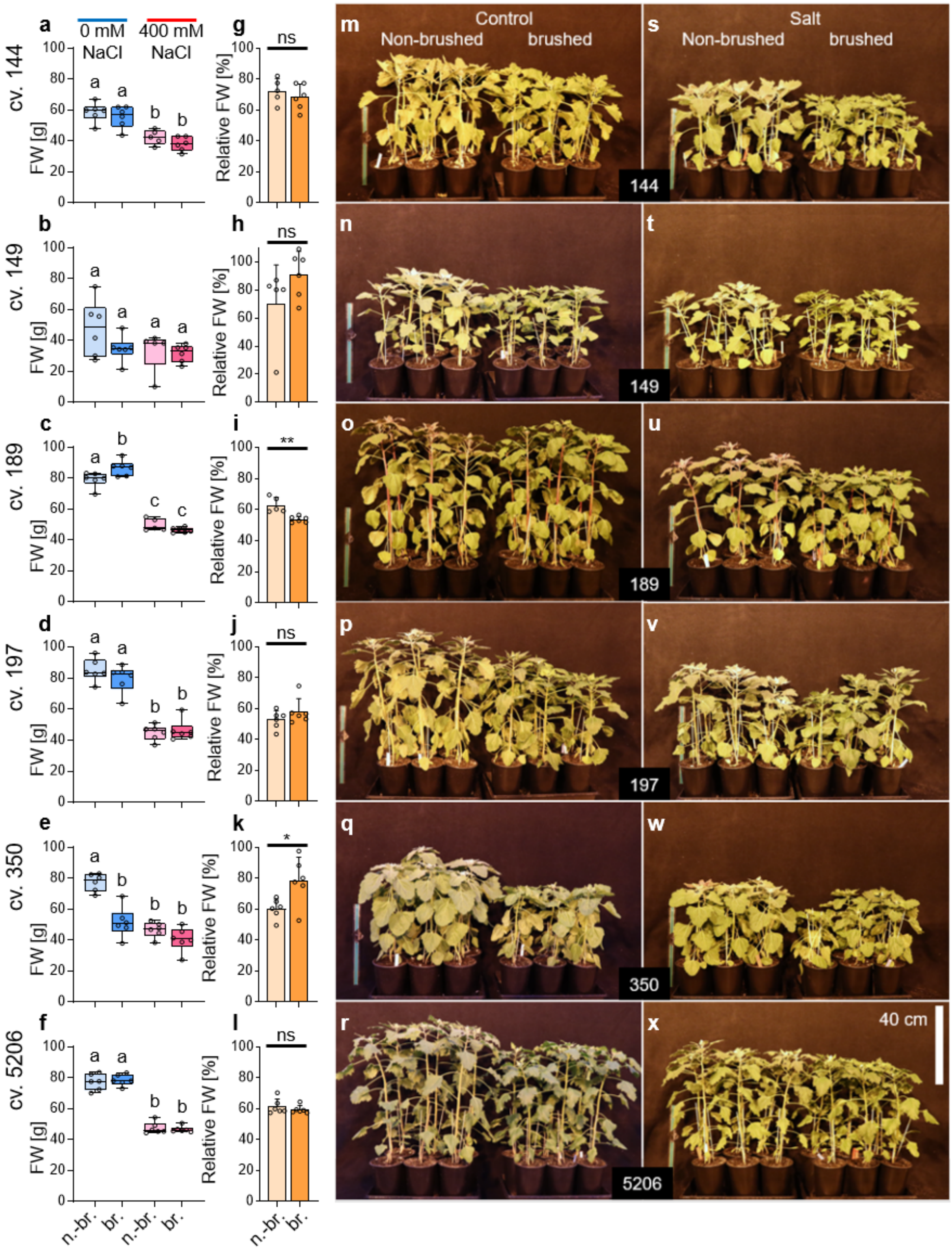

0 mM NaCl
400 mM NaCl
cv. 144
cv. 149
cv. 189
cv. 197
cv. 350
cv. 5206
FW [g]
Relative FW [%]
ns
**
*
Control
Salt
Non-brushed
brushed
144
149
189
197
350
5206
40 cm
n.-br.
br.

Figure S6. Mechanical removal of EBCs does not affect growth at 400 mM NaCl in a variety of quinoa cultivars. Quinoa plants of different cultivars were irrigated with tap water for 2 weeks and then side branches were removed and all EBCs (epidermal bladder cells) were gently removed with a cosmetic brush from one set of plants (br. = brushed), while EBCs were not brushed from the other set of plants (n.-br. = non-brushed). During the following 5 weeks of 400 mM NaCl irrigation, EBCs of newly emerging leaves on the br. plants were removed three times a week. While brushed and non-brushed plants receiving 400 mM NaCl did not show any differences in biomass acquisition, cv. 189 showed increased and cv. 350 showed decreased biomass in plants irrigated with tap water (0 mM) (**a**-**f**). Differences in growth of control plants led to altered relative fresh weights (FW) in these cultivars due to salt treatment, while in the other cultivars relative biomass production was not altered due to brushing (**g**-**l**). Images show 7-week-old plants after 0 (**m**-**r**) and 400 mM NaCl (**s**-**x**) treatment. Scale bar, 40 cm. The boxes extend from the 25th to 75th percentiles, with whiskers ranging from minimum to maximum values. The horizontal lines inside the boxes indicate the medians ($n = 6$). Different letters indicate significant differences ($P < 0.05$) from one-way ANOVA followed by Tukey's multiple comparison test. Bar graphs with mean values ± s.d. $n = 6$ (ns: p value >0.05, *: p value <0.05 and **: p value <0.01). Individual values are presented as open circles.

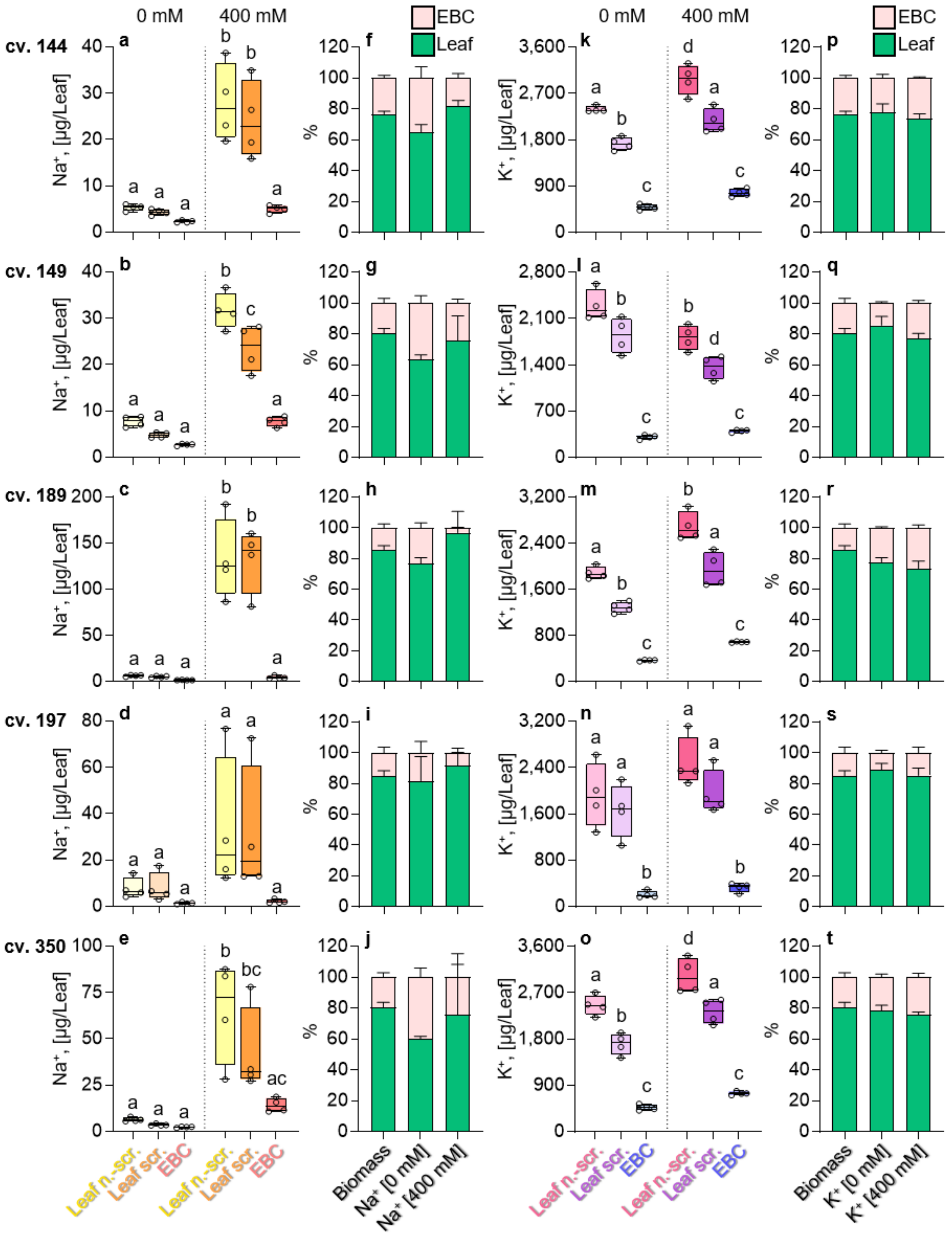

Figure S7. EBCs are not able to remove relevant amounts of Na⁺ from the leaf in cultivars of quinoa with varying tolerance and EBC fractions. With the exception of *C. quinoa*

cultivar cv. 149, the low amount of Na^+ in the EBC (epidermal bladder cells) fraction of the leaf was not sufficient to remove significant amounts of salt from the leaf (**a**-**e**). Therefore, EBCs should not be classified as salt dumps. Interestingly, the distribution of Na^+ was only shifted to EBCs when plants were not irrigated with additional NaCl in the irrigation water (**f**-**j**). However, in most cultivars, significant amounts of K^+ were found in EBCs (**k**-**t**). The boxes extend from the 25th to 75th percentiles, with whiskers ranging from minimum to maximum values. The horizontal lines in the boxes indicate the medians ($n = 4$). Different letters indicate significant differences ($P < 0.05$) from a one-way ANOVA followed by a Tukey's multiple comparison test. Bar graphs show relative distributions, Error bars represent ± SD. Individual values are presented as open circles. Abbreviations: Leaf n.-scr., non-scraped leaf; Leaf scr., scraped leaf.

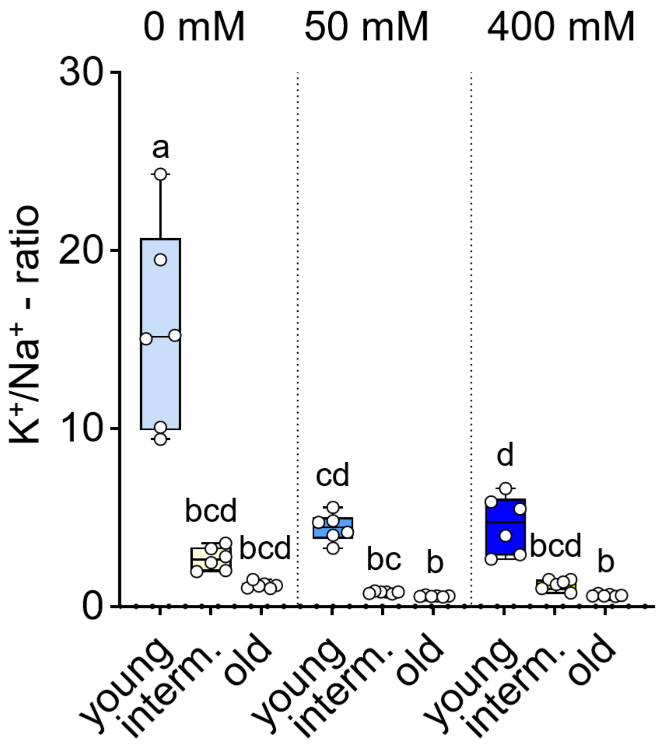

Figure S8. High K^+/Na^+ ratios only in young leaves of quinoa during salt stress. Unequal distribution of Na^+ and K^+ results in the highest K^+/Na^+ ratios in the young leaves of the WT. The boxes extend from the 25th to 75th percentiles, with whiskers ranging from minimum to maximum values. The horizontal lines in the boxes indicate the medians (n = 6). Different letters indicate significant differences ($P < 0.05$) from one-way ANOVA followed by Tukey's multiple comparison test. Individual values are presented as open circles.

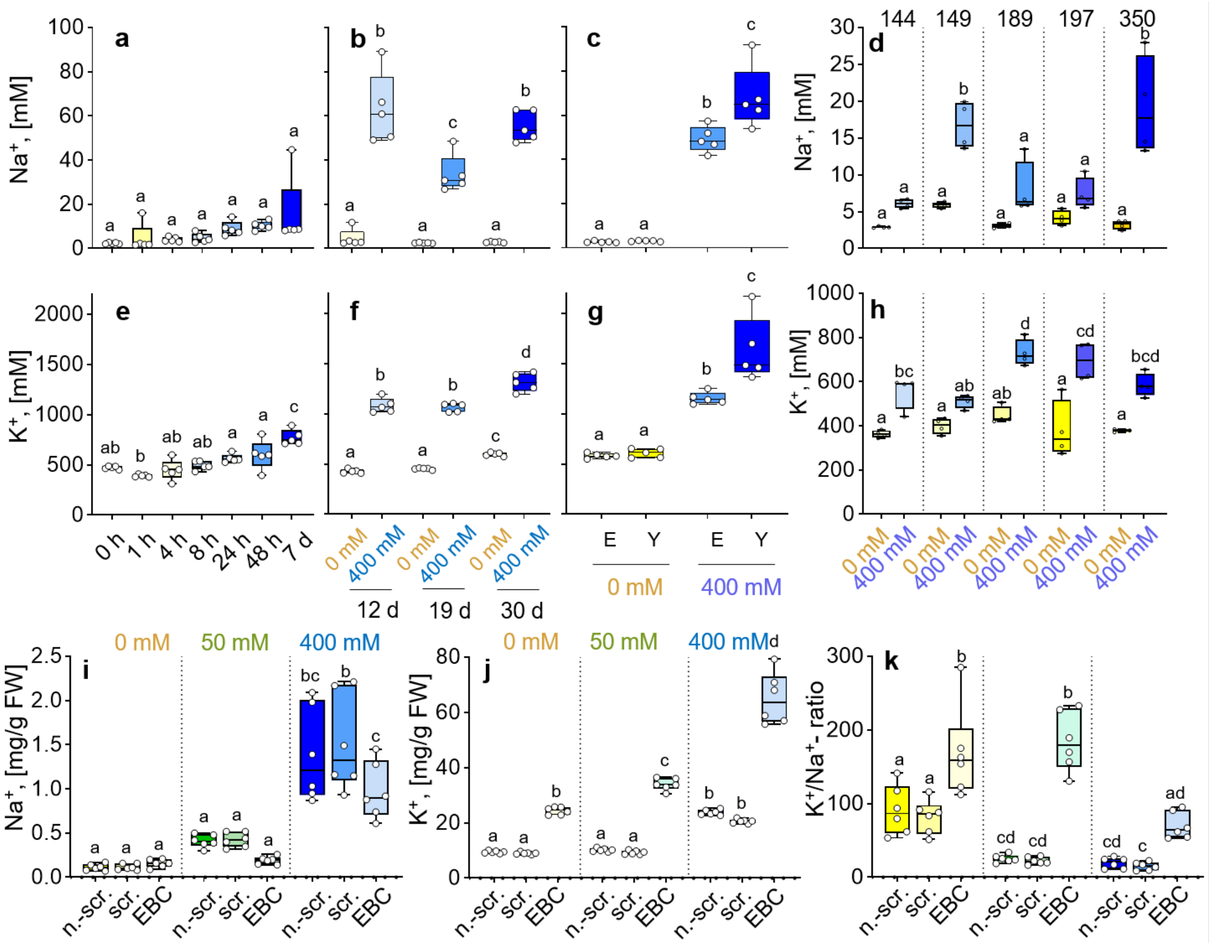

Figure S9. EBCs of quinoa accumulate K^+ over Na^+. Na^+ concentrations in EBCs increased but remained low after a salt shock with 300 mM NaCl (**a**) and prolonged salt irrigation (400 mM NaCl) (**b**). This pattern was unaffected by the age of the leaf (**c**) and the cultivar (after 5 weeks of 400 mM NaCl) (**d**). By contrast, high K^+ concentrations were measured already under control conditions and increased further when plants were irrigated with NaCl (**e–h**). While Na^+ concentrations were found to be lower in EBCs than in the leaf (**i**), EBC K^+ concentration exceeded that of the leaf (**j**), resulting in high K^+/Na^+ ratios in these cells (**k**). E = emerging; Y= young. The boxplots extend from the 25th to 75th percentiles, with whiskers ranging from minimum to maximum values. The horizontal lines in the boxes indicate the medians (n = 5; for cultivars n = 4). Different letters indicate significant differences ($P < 0.05$) from a one-way ANOVA followed by a Tukey's multiple comparison test. Individual values are presented as open circles.

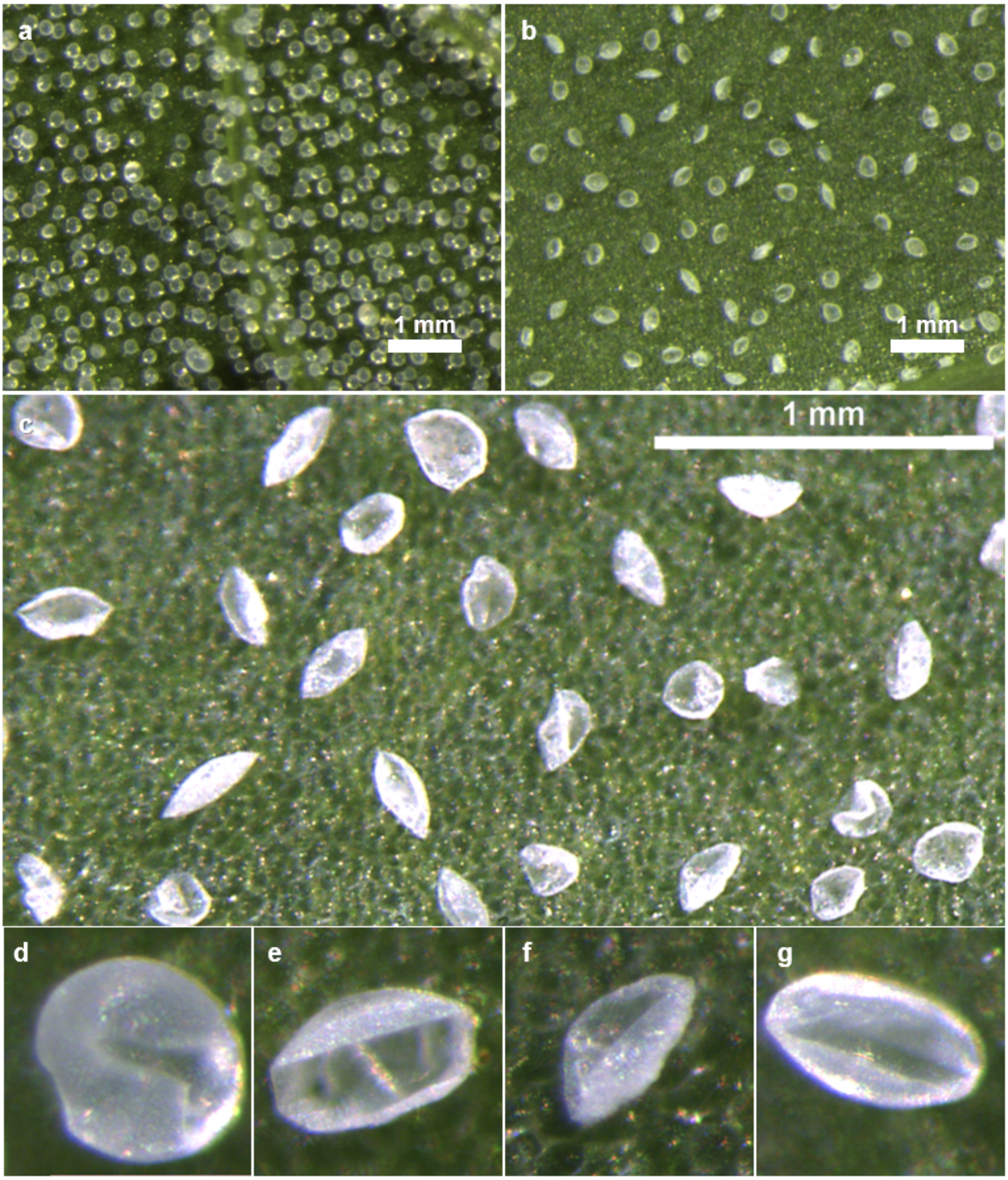

139

140 **Figure S10. Microscopy analysis of EBCs of quinoa.** EBCs are turgid on young leaves (**a**)
141 but deflate as leaves mature (**b**). Overview (**c**) and close-up (**d–g**) images of deflated EBCs.
142 Scale bars, 1 mm.
